# Trauma-Informed Care Interventions Used in Pediatric Inpatient or Residential Treatment Mental Health Settings and Strategies to Implement Them: A Scoping Review

**DOI:** 10.1177/15248380231193444

**Published:** 2023-09-11

**Authors:** Yehudis Stokes, Krystina B. Lewis, Andrea C. Tricco, Erin Hambrick, Jean Daniel Jacob, Melissa Demery Varin, Justine Gould, Dhiraj Aggarwal, Paula Cloutier, Catherine Landriault, Stephanie Greenham, Michelle Ward, Allison Kennedy, Jennifer Boggett, Roxanna Sheppard, David Murphy, Marjorie Robb, Hazen Gandy, Sonia Lavergne, Ian D. Graham

**Affiliations:** 1 University of Ottawa, ON, Canada; 2 Children’s Hospital of Eastern Ontario (CHEO), Ottawa, Canada; 3 CHEO Research Institute, Ottawa, Canada; 4 Princess Margaret Cancer Centre, Toronto, ON, Canada; 5 University of Ottawa Heart Institute, ON, Canada; 6 Ottawa Hospital Research Institute, ON, Canada; 7 Queen’s University, Kingston, ON, Canada; 8 University of Toronto, ON, Canada; 9 Li Ka Shing Knowledge Institute, St. Michael’s Hospital, Unity Health Toronto, ON, Canada; 10 University of Missouri-Kansas City, MO, USA; 11 Royal Ottawa Mental Health Centre, ON, Canada

**Keywords:** cultural contexts, treatment/intervention, child abuse, vicarious trauma

## Abstract

Trauma-informed care (TIC) is an approach to care emerging in research and in practice that involves addressing the needs of individuals with histories of trauma. The aim of this scoping review was to examine the current literature relating to TIC interventions used in pediatric mental health inpatient and residential settings. We sought to answer the following two research questions: (a) What are the TIC interventions used in pediatric inpatient and residential treatment mental healthcare settings and what are their components? and (b) What are the implementation goals and strategies used with these TIC interventions? We conducted this scoping review according to JBI (formerly Joanna Briggs Institute) methodology for scoping reviews. We included any primary study describing a TIC intervention that was implemented at a specific site which identified and described implementation strategies used. Of 1,571 identified citations and 54 full-text articles located by handsearching, 49 met the eligibility criteria and were included, representing 21 distinct TIC interventions. We present the reported aim, ingredients, mechanism, and delivery (AIMD) of TIC interventions as well as the implementation goals and strategies used, which varied in detail, ranging from very little information to more detailed descriptions. In the context of these findings, we emphasize the complexity of TIC and of TIC interventions, and the importance of identifying and clearly reporting TIC intervention goals, intervention details, and implementation strategies. We suggest applying intervention frameworks or reporting guidelines to support clear and comprehensive reporting, which would better facilitate replication and synthesis of published TIC interventions.

## Background

### Trauma-Informed Care

“Trauma-informed care” (TIC) is a philosophy that involves addressing the needs of individuals with histories of trauma, whether they are the ones seeking care, or providing care (Fallot & Harris, 2009). Generally agreed upon assumptions of TIC include the four R’s: Realizing the prevalence of trauma, Recognizing manifestations of trauma, Responding appropriately to trauma, and Resisting Re-traumatization. Additionally, many apply the seven principles of TIC: safety (physical and psychological), trustworthiness and transparency, peer support, collaboration and mutuality, empowerment, voice and choice, and cultural, historical, and gender considerations (e.g., Fallot & Harris, 2009; Substance Abuse and Mental Health Services Administration (SAMHSA), 2014). Yatchmenoff et al. (2017) reduce these principles to three common elements: safety, power, and self-worth. For an organization to be trauma-informed, the culture of the organization must reflect the above principles in every interpersonal contact, setting, and relationship, as perceived by both staff and consumers (Fallot & Harris, 2009). Furthermore, it is important that organizations consider every patient and staff member as though they may have trauma histories, an approach known as ‘universal precautions’ (Elliott et al., 2005; Harris & Fallot, 2001).

### Trauma-Informed Care in Pediatric Mental Health Settings

Extensive research has linked childhood exposure to trauma and maltreatment with higher rates of physical and mental disorders and healthcare use (e.g., Chu, 2011; Cuijpers et al., 2011; Felitti, 2009; Felitti et al., 1998; Huffhines et al., 2016). A chart review of pediatric psychiatric inpatient hospitalizations was conducted over a 10-month period (Keeshin et al., 2014). The authors found that children and adolescents with a history of maltreatment were more likely to be diagnosed with multiple disorders than youth without a history of trauma (physical abuse adjusted odds ratio = 1.93, p < 0.001; sexual abuse adjusted odds ratio = 2.97; p < 0.001; Keeshin et al., 2014). These authors identified that physical and sexual abuse histories in patients were also independently associated with increased length of stay by 2.3 days (F(3, 1075) = 9.2, p < 0.001; Keeshin et al., 2014), all highlighting the need for trauma-informed services in pediatric mental health settings. Additionally, there may be unique factors to consider in addressing trauma in children and youth within pediatric-specific contexts, such as developmental considerations and relationships between youth and adult caregivers (Lowenthal, 2020).

We identified three recent reviews of TIC interventions (TICI) in pediatric mental health settings (Bailey et al., 2019; Bryson et al., 2017; Lowenthal, 2020). Bailey et al. (2019) systematically reviewed the empirical evidence for organization-wide TICI in out-of-home care published from 2002 to 2017. The authors identified three TICI across seven studies: Attachment Regulation and Competency framework (3 studies), Children and Residential Experiences programme (1 study), and The Sanctuary Model (3 studies) (Bailey et al., 2019).

Bryson et al. (2017) conducted a realist review of implementation strategies of organization-wide TICI in child and adolescent inpatient psychiatric and residential settings including literature from 2000 to 2015. Across 13 articles, they identified five primary factors relating to successful TIC implementation: (a) senior leadership prioritizing TIC, (b) aligning organizational policies and practices, formal and informal, with the principles of TIC, (c) listening to patients’ and families’ experiences, needs, and priorities, (d) supporting staff through training and providing ongoing supervision, coaching, and debriefing, and (e) reviewing data and outcome indicators to foster continuous improvement.

Finally, Lowenthal (2020) performed a scoping review to describe the characteristics of TIC implementation research in child and youth serving sectors. Consistent with the previous reviews, Lowenthal (2020) included only articles that reported results of the TIC implementation initiative. They included 54 articles published between 2004 and 2019, 17 of which were identified as psychiatric inpatient or residential treatment settings. Lowenthal (2020) classified implementation interventions as limited change initiatives (e.g., one-off training with little follow-up), moderate change initiatives (e.g., using a few different types of initiatives over a moderate period of time), and comprehensive change initiatives (e.g., using a multifaceted approach over longer periods of time to support changes in the organizational culture, structure, and policies). This review highlighted the lack of details available in the included studies pertaining to the actual TICI and their implementation strategies.

These three reviews provide preliminary evidence to support organizations wishing to implement TICI in pediatric mental health settings. Yet, the theoretical and practical aspects of TICI remain unclear, particularly as they relate to how TICI are implemented or operationalized in pediatric settings. Authors of these reviews limited their literature searches to articles which reported evaluative results (of the TICI and/or the implementation strategies), and none reported, in detail, the components of each of the TICI nor the implementation strategies used at each site. The most recent review by Lowenthal (2020) provided an overview of the methodological approaches used, geographical locations, and service sectors related to each intervention, however, lacked descriptive details pertaining to the TICI (i.e., what was the actual TIC intervention). The author classified the scope of the TICI implementation initiatives, yet did not clearly describe the criteria, nor the basis for the criteria, for the classification decisions. Furthermore, the article did not include a comprehensive summary of the implementation strategies used within each study.

## Research Questions

Within this review, we sought to answer the following research questions: What are the TICI used in pediatric inpatient and residential treatment mental healthcare settings and what are their components? What are the implementation goals and strategies used with these TICI?

## Method

### Protocol, Registration, and Reporting Methods

This scoping review was conducted according to JBI (formerly Joanna Briggs Institute) methodology for scoping reviews (Peters et al., 2020) guided by an *a priori* protocol in Open Science Framework (Stokes et al., 2020). We report this review in accordance with the Preferred Reporting Items for Systematic Reviews and Meta-Analyses extension for scoping reviews (PRISMA-ScR) (checklist in Appendix A) (Tricco et al., 2018).

### Search Strategy

**Figure 1.**
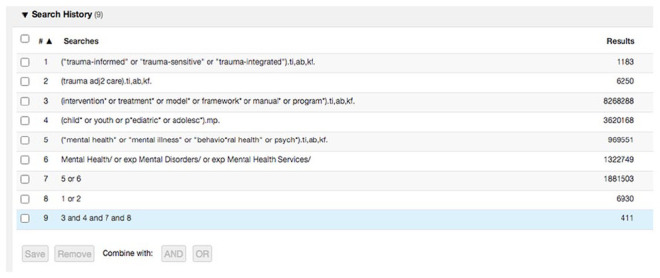
Search strategy in Medline.

We applied the three-step search strategy recommended by JBI for scoping reviews (Peters et al., 2020). First, we conducted a pilot search of one of the relevant databases (MEDLINE), designed by the first author (YS) in collaboration with a library scientist (MS). YS subsequently performed an analysis of the text words contained in the titles and abstracts of the identified papers and of the respective descriptive index terms. Second, YS designed a second search, including all the identified keywords and index terms. We performed this search in CINAHL, MEDLINE, and PsycINFO on September 2, 2020. A library scientist (MS) peer reviewed the search strategy in accordance with the Peer Review of Electronic Search Strategies (PRESS) guidelines (Mcgowan et al., 2016). Third, YS screened the reference lists of all included studies for additional studies. The full electronic search strategy for Medline is found in Figure 1 and all search strategies are available through the Open Science Framework (Stokes et al., 2020).

### Eligibility Criteria

**Population.** We included pediatric patients under the age of 18, their families, and/or staff (including, but not limited to, healthcare providers) and excluded infant populations under age two. **Intervention.** We included clinical interventions described by authors as “trauma-informed,” “trauma-integrated,” or “trauma-sensitive.” These interventions (including models, programs, and initiatives) may be targeted to patients, their families, and/or staff members. We excluded interventions for individuals with a diagnosis of post-traumatic stress disorder (PTSD) or designed to process a trauma narrative because TICI are intended to be inclusive and applicable to individuals without a known history of trauma. We also excluded interventions that were not milieu-based interventions (e.g., stand-alone therapeutic groups). We excluded articles that did not include a description of implementation strategies used, or only described the hypothetical implementation of the TICI. **Context.** We included pediatric inpatient or residential treatment mental healthcare settings and excluded juvenile justice, secure settings, and school settings that were not clearly described as a mental health treatment program. **Study Type.** We included any primary study describing a TICI that was implemented at a specific site which identified and described implementation strategies used. Related reviews were excluded, yet their reference lists were screened. **Language.** We only included studies published in English or French. **Publication date.** We limited the search to literature from 1995 onwards because TIC is a concept that was first used in approximately 2001 by Harris and Fallot (2001). **Publication status.** We included peer reviewed articles and excluded grey literature including theses.

### Study Selection

YS uploaded all citations and abstracts to Covidence systematic review software (Veritas Health Innovation, Melbourne, Australia; available at www.covidence.org) and removed duplicates. Two reviewers (YS and MDV) independently screened studies for eligibility using Covidence. We initially conducted a pilot test by screening a random sample of 25 titles/abstracts for inclusion, and then met to discuss discrepancies and to modify the inclusion criteria, if required. Articles were excluded if both reviewers agreed to exclude them, otherwise, they proceeded to the next step of full-text screening. Reviewers met to resolve conflicts, and a third team member (EH or IDG) resolved conflicts as needed. The reviewers then independently screened the remaining full texts in a similar fashion. Articles were excluded if both reviewers agreed to exclude them, and reasons for exclusion were documented. Systematic reviews and scoping reviews meeting first-level screening were retained and YS screened their reference lists for eligible studies.

#### Data extraction

Two reviewers (YS and JG or MDV or MH) independently extracted study details from the included articles into a template form in REDcap (Research Electronic Data Capture; Harris et al., 2009). We pilot-tested the extraction process as follows: each reviewer independently extracted the data for six articles and then met to compare and reach consensus. At that time, we discussed and revised the forms prior to continuing with the rest of the data extraction. Any disagreements between reviewers’ extractions were resolved through discussion, or with a third reviewer when needed.

We extracted the following data items: study characteristics (first author, study year, year of publication, journal, language, country, corresponding author, study type, study funding, study limitations); study authors’ definition of TIC (verbatim) and reported theoretical underpinnings of TIC; study aims; setting details (type of setting, treatment population of setting, location, urban/ rural location); descriptors of TIC intervention participants, including patients, families, full-time equivalent (FTE) of staff; intervention and implementation strategy details, as described below.

We used the AIMD framework (Aims, Ingredients, Mechanism, Delivery; see Table 1 for definitions) (Bragge et al., 2017) to guide the extraction of information about the TICI and implementation strategies using the following process: Two reviewers (YS and JG) independently extracted the data from each article into Microsoft Word tables and extracted in the same manner any specified goals of the implementation strategies, and any information regarding theory or frameworks used to guide the implementation of the TIC intervention. We resolved discrepancies through consensus.

**Table 1.** AIMD Definitions.

Component	Description	Definition and Considerations
Aims	What will be achieved and for whom? (*what are the targets or goals of the intervention*)	This component relates to the objective and outcome of the intervention. Based on your endpoint, what are you measuring in whom?
Ingredients	What comprises the intervention? (*what are the essential components of intervention*)	These are the observable, replicable, and irreducible aspects of the intervention.
Mechanism	How do you propose the intervention will work? (*how does the intervention work; what is the theoretical rationale for the intervention*)	This refers to the pathways or processes by which it is proposed that an intervention effects change or which change comes into effect.
Delivery	How will you deliver the intervention? (*how is the intervention delivered to clients/patients/caregivers*)	This encompasses logistical and practical information pertaining to intervention delivery, including mode (e.g. video, brochure); level (e.g. individual, team, population); dose, frequency, intensity; who’s delivering; and size of target group.

*Source*. Adapted from Bragge et al., 2017.

### Data Analysis

#### TICI

We aggregated multiple reports on the same TICI, so that each intervention was a unit of interest in the review. We used synthesis tables and descriptive summaries to report on the article characteristics, the TICI characteristics, and the implementation strategies. Two reviewers (YS and JG) independently and (subsequently) collaboratively distilled and summarized the AIMD tables. We compared similarities and differences between the reported interventions and performed a high-level content analysis of TICI objectives.

#### Goals of implementation strategies

We categorized the goals of the implementation strategies based on the Canadian Institutes of Health Research (CIHR, 2012) categories for knowledge translation (KT) planning goals.

#### Implementation strategies

We categorized implementation strategies based on the 73 implementation categories defined by the Expert Recommendations for Implementing Change (ERIC; Powell et al., 2015), incorporating the updates recommended for existing category names and definitions as well as three additional categories (Perry et al., 2019), and using Waltz et al. (2015) thematic classification system. YS and JG independently applied the ERIC strategies and implementation goal categories, and discrepancies were resolved with IDG and KBL. We inductively identified an additional category named (77: “Align with organizational or government mandate”) to incorporate a strategy that was not otherwise captured. The four additional categories (three added by C.K. Perry and colleagues, one added by our team) were not included in Waltz and colleagues’ thematic classification system and were therefore labeled as “not categorized.” Throughout this article we will refer to the ERIC implementation categories as the ERIC implementation strategies. We tabulated frequency counts of the ERIC implementation strategies, the ERIC thematic classifications, and the implementation goal strategies and presented summaries in table formats. All categorical synthesis used simple content analysis, in accordance with the JBI guide.

## Results

**Figure 2.**
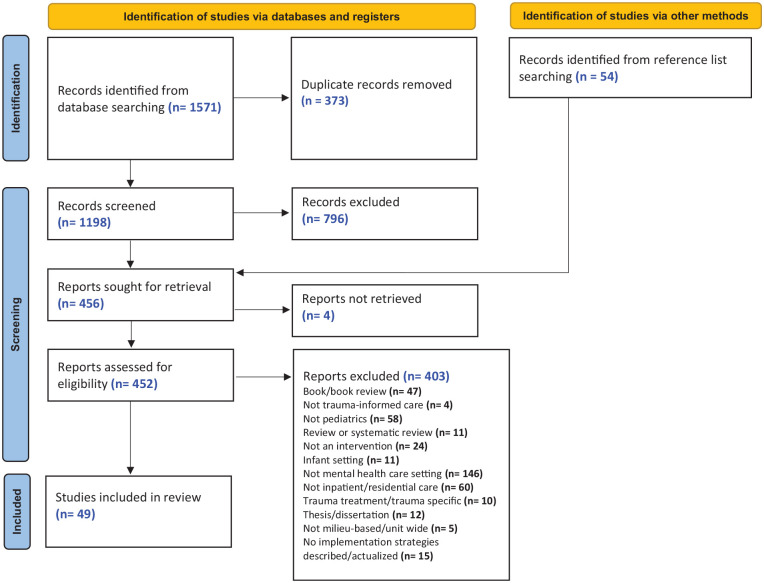
PRISMA flowchart.

Of 1,571 identified citations and 54 full-text articles located by handsearching, 49 met the eligibility criteria and were included (Figure 2). The earliest article identified was published in 2003 (regarding the Sanctuary Model), with more than one third (n = 19, 39%) published since 2016. In Table 2 we present the characteristics of the 49 included articles. Most articles (n = 41, 84%) were conducted in the USA, while a few were based in Canada (n = 3, 6%) and Australia (n = 4, 8%), and one article (2%) included sites from Canada, Scotland, and USA. Most articles (n = 39; 80%) contained non-experimental study designs, and 20% of articles (n = 10) used experimental or quasi experimental designs. Most intervention sites were residential treatment/congregate care treatment settings (n = 28, 57%), followed by inpatient psychiatric hospital settings (n = 9, 18%). Two articles (4%) reported on both inpatient and residential treatment sites, five articles (10%) were of statewide initiatives, and three articles (6%) were set in child welfare systems that incorporated residential treatment. One article (2%) took place in a mental health treatment unit within a juvenile justice setting, and one (2%) was public health unit intervention which included staff from pediatric psychiatric facilities.

**Table 2.** Study Characteristics (*n* = 49) Sorted by Intervention (*n* = 21).

Intervention Name	Author (Year)	Country	Setting	Article Type	Funding (F); Conflict of Interest (CoI)*
Attachment, Regulation and Competency (ARC) *n* = 4					
Project Penguin informed by ARC and Positive Behavioural Interventions and Supports (PBIS)	Brend et al. (2020)	Canada	Residential treatment centres in child welfare	Mixed methods (non-controlled before and after and qualitative)	F: Government; CoI:RN
Building Communities of Care (BCC)	Forrest et al. (2018)	USA (author affiliation)	6 residential treatment programs	Non-controlled before and after	F: NR; CoI:RN
ARC; Grow strong/stepping stones	Hodgdon et al. (2013)	USA	2 residential treatment programs serving female youth	Non-controlled before and after	F:Government; CoI:NR
Trauma-Informed Care (TIC) training informed by the Substance Abuse and Mental Health Services Administration (SAMHSA) and ARC	Matte-Landry and Collin-Vézina (2022)	Canada	44 residential units for children and youth	Interrupted time series	F: Government & Academic; CoI: RN
Child Adult Relationship Enhancement (CARE) *n* = 1					
Child Adult Relationship Enhancement (CARE)	Gurwitch et al. (2016)	USA	State of Delaware Division of Prevention and Behavioral Health Services (DPBHS), including youth inpatient units	Case study	F: Foundation, Government; CoI:RN
Children and Residential Experiences (CARE) *n* = 1					
Children and Residential Experiences (CARE)	Izzo et al. (2016)	USA	11 group care agencies serving youth from child welfare	Interrupted time series	F: Foundation; CoI:RY
Collaborative Problem Solving (CPS) *n* = 5					
CPS	Greene et al. (2006)	USA	1 inpatient child psychiatric unit	Non-controlled before and after	F:NR; CoI:NR
CPS	Martin et al. (2008)	USA	1 child psychiatric inpatient setting	Non-controlled before and after	F:Foundation; CoI:RN
CPS	Pollastri et al. (2016)	USA	1 youth mental health agency providing residential treatment	Interrupted time series	F:NR; CoI:NR
“Major Change” including CPS	Regan (2010)	USA	1 inpatient child psychiatric unit	Case series	F:NR; CoI:RN
Child and Family Centered Care (CFCC): Including collaborative CPS	Regan et al. (2017)	USA	1 inpatient child psychiatric unit	Non-controlled before and after	F:Government, Acadmic; CoI:NR
Devereux’s Safe and Positive Approaches (SPA) *n* = 1					
SPA	Russell et al. (2009)	USA	Programs offering residential treatment to children and adolescents	Prospective cohort study	F:NR; CoI:NR
EQ2: Empowering direct care staff to build trauma-responsive communities for youth *n* = 1					
EQ2	Griffing et al. (2021)	USA	1 short-term crisis stabilization unit and 2 residential treatment centres for adolescents	Mixed methods (non-controlled before and after and qualitative)	F:Foundation; CoI:NR
NASMHPD Six Core Strategies *n* = 5					
Six Core Strategies	Azeem et al. (2011)	USA	State psychiatric hospital with child and adolescent unit	Non-controlled before and after	F:NR; CoI:RN
Six Core Strategies	Azeem et al. (2015)	USA	Pediatric psychiatric hospital	Non-controlled before and after	F:NR; CoI:NR
“Broad TIC program” based on Six Core Strategies and Risking Connections	Barnett et al. (2018)	USA	Youth residential treatment center	Non-controlled before and after	F:Foundation; CoI:NR
Six Core Strategies, Building Bridges Initiative (BBI)	Caldwell et al. (2014)	USA	3 programs: (a) Pediatric psychiatric hospital, (b) secure residential treatment centre, (c) residential services	Non-controlled before and after	F:NR; CoI:RN
TIC Program based on the Six Core Strategies	Hale and Wendler (2023)	USA	Inpatient psychiatric hospital, which cares for children and adolescents	Non-controlled before and after	F:RN; CoI:RN
Neurosequential Model of Therapeutics (NMT) *n* = 1					
NMT	Hambrick et al. (2018)	USA/Canada/Scotland	10 residential and day treatment settings for adolescents	Interrupted time series	F:Foundation, Gov, Private; CoI:RN
Patient-Focused Intervention (PFI) Model *n* = 1					
PFI Model	Goetz and Taylor-Trujillo (2012)	USA	Adolescent residential treatment unit in an inpatient psychiatric facility	Non-controlled before and after	F:RN; CoI:RN
Risking Connection (RC) and Restorative Approach (RA) *n* = 2					
RC and RA	Baker et al. (2018)	Canada	1 residential youth services division	Mixed methods (non-controlled before and after and qualitative)	F: Academic, Government; CoI:NR
RC and RA	S. M. Brown et al. (2012)	USA	5 child congregate care agencies	Non-controlled before and after	F:NR; CoI: RY
Sanctuary Model *n* = 11					
Sanctuary Model	Bloom (2003)	USA	2 residential treatment centres for children	Case series	F:Government; CoI:NR
Sanctuary Model	Bloom et al. (2003)	USA	2 Residential treatment centres and 1 group home for children and adolescents	Case series	F:NR; CoI:NR
Sanctuary Model	Clarke (2012)	Australia	Residential care programs for children and young people	Case study	F:NR; CoI:NR
Sanctuary Model	Esaki et al. (2014)	USA	Child welfare agency that offers residential care	Cross-sectional	F:NR; CoI:NR
Sanctuary Model	Farragher and Yanosy (2005)	USA	Residential and day treatment program for youth	Case study	F:NR; CoI:NR
Sanctuary Model	Leigh-Smith and Toth (2014)	Australia	Residential care programs for children and young people	Case study	F:NR; CoI:NR
Sanctuary Model	McCorkle and Peacock (2005)	USA	1 residential treatment facility for children and adolescents	Case study	F:NR; CoI:NR
Sanctuary Model	Rivard et al. (2003)	USA	17 residential treatment units across 3 programs	Cluster randomized-controlled trial	F:Government; CoI:NR
Sanctuary Model	Rivard (2004)	USA	17 residential treatment units across 3 programs	Cluster randomized-controlled trial	F:Government; CoI:NR
Sanctuary Model	Rivard et al. (2004)	USA	17 residential treatment units across 3 programs	Mixed methods (cluster randomized-controlled trial and qualitative)	F:Government; CoI:NR
Sanctuary Model	Rivard et al. (2005)	USA	8 residential treatment units for youth	Non-controlled before and after	F:Government; CoI:NR
Sensory integration initiatives *n* = 5					
Trauma-Informed Care (TIC) and Ayres Sensory Integration Training	Denison et al. (2018)	USA	Residential treatment centre for adolescents	Non-controlled before and after	F:NR; CoI:NR
Massachusetts R/S prevention initiative to promote strength-based care	Lebel et al. (2004)	USA	State-wide child and adolescent inpatient units	Non-controlled before and after	F:Government; CoI:NR
Massachusetts State R/S prevention initiative: integrating sensory and trauma-informed interventions	Lebel and Champagne (2010)	USA	State-wide including child and adolescent mental health facilities	Non-controlled before and after	F:NR; CoI:NR
Sensory modulation and trauma-informed-care	McEvedy et al. (2017)	Australia	State-wide, 19 area mental health services	Qualitative	F:Government; CoI:RN
Sensory room/occupational therapy (OT) consultation/Sensory Motor Arousal Regulation Treatment (SMART)	Warner et al. (2013)	USA	Residential treatment sites for adolescents	Case series	F: Source not specified; CoI:NR
Structured Psychotherapy for Adolescents Responding to Chronic Stress (SPARCS) *n* = 1					
SPARCS	Habib et al. (2013)	USA	1 residential program for adolescents	Non-controlled before and after	F:NR; CoI:NR
Trauma Affect Regulation: Guide for Education and Therapy (TARGET) *n* = 1					
TARGET and environmental modifications	Marrow et al. (2012)	USA	2 mental health units in juvenile justice settings	Non-randomized cluster-controlled trial	F:NR; CoI:NR
Trauma-Informed Psychiatric Residential Treatment (TI-PRT) *n* = 1					
TI-PRT	Boel-Studt (2017)	USA	Psychiatric residential facilities of a large Behavioral Health Agency	Historically controlled cohort study	F:RN; CoI:RN
Trauma-Systems Therapy (TST) *n* = 3					
TST	A. D. Brown et al. (2013)	USA	3 child and adolescent residential treatment centres	Case series	F:NR; CoI:NR
TST	Murphy et al. (2017)	USA	1 private child welfare system	Interrupted time series	F:Foundation; CoI:RN
TST/bridging the way home initiative	Redd et al. (2017)	USA	1 private child welfare system	Mixed methods (interrupted time series and qualitative)	F:Foundation; CoI:RN
Uncategorized TIC programs *n* = 5					
Gender-specific and trauma-informed training curriculum	Crable et al. (2013)	USA (author affiliation)	Residential group care facility	Non-controlled before and after	F:NR; CoI:NR
Trauma-informed approach (TIA)	Craig and Sanders (2018)	USA	Behavioral healthcare facility	Non-controlled before and after	F:NR; CoI:RN
A trauma-informed care program	Jacobowitz et al. (2015)	USA	Psychiatric hospital providing short-term acute care (including child and youth)	Cross-sectional	F:NR; CoI:RN
Trauma-informed child welfare service (CWS)	Lang et al. (2016)	USA	State-wide including child and adolescent mental health facilities	Non-controlled before and after	F:Government; CoI:RN
TIC training program	Williams and Smith (2017)	Australia	Public mental health, including staff from pediatric mental health facilities	Cross-sectional	F:Government; CoI:NR

*Note*. Conflicts: NR = No conflict reported; RN = reported no conflicts; RY = reported conflicts.

### TIC Interventions

We grouped together articles reporting on identical sites, and subsequently collapsed all articles reporting on the same intervention, resulting in 21 distinct TICI (Table 2). Some interventions were described by multiple articles, including the Sanctuary Model (11 articles), Collaborative Program Solving (CPS; five articles), the National Association of State Mental Health Program Directors (NASHMHPD) Six Core Strategies (five articles), Attachment, Regulation and Competency (ARC; four articles), Trauma Systems Therapy (TST; three articles), and Risking Connection and Restorative Approach (RC and RA; two articles). Five articles described initiatives that were sensory-integration based. There were five additional TICI that did not fall into the categories above. **TIC intervention aims.** In Table S1 we present the AIMD (Aims, Ingredients, Mechanism, Delivery) of each TICI (n = 21) as reported in the included articles. Many articles did not explicitly state the intervention aims/goals, making it difficult for reviewers to identify and extract. We identified six overarching themes in the reported aims of the interventions which we sequenced from narrow to broader range in focus: (a) to reduce incidence of restraints, seclusions, and critical events (eight interventions), (b) to change staff attitudes and skills or behaviors towards patients (11 interventions), (c) to increase or change available assessments and treatments or to coordinate care (10 interventions), (d) to support staff wellness or increase staff wellness capacities (four interventions), (e) to increase patient wellness capacities or improve patient outcomes (eight interventions), and (f) to change culture (12 interventions). We present the TICI aims in Table 3. Most articles reported multiple TICI aims. Two TICI (three articles) had no explicitly reported aims. Overall, the intervention aims were varied with no single common aim amongst the 21 TICI. However, some aims were more common than others with more than half of the included interventions described as aiming to change the organizational culture. **TIC intervention ingredients.** The reported essential components or ingredients of the TICI varied in detail, ranging from very little information (nine of the 49 included articles) to more detailed descriptions of the intervention ingredients. **TIC intervention mechanisms.** Most TICI (18, 86%) included some description of their underlying mechanism and/or theoretical basis (i.e., Risking Connection being based on the constructivist self-development theory (S. M. Brown et al., 2012); the mechanism of TARGET is “to maximize a person’s awareness of the present moment, thereby reducing mental health symptoms commonly associated with trauma, such as rumination, panic, or dissociation” (Marrow et al., 2012; p. 260)). **TIC intervention delivery.** Details related to the delivery of the TICI also varied, with 15 articles not reporting any detail of intervention delivery and only describing the implementation of the intervention, (i.e., training, without explaining how the actual TICI would be delivered in practice). Across the AIMD analysis (see Table S1), several studies highlighted elements that were related to pediatric context. Namely, an emphasis on parent or caregiver involvement within the essential ingredients or the delivery of the TICI (17 articles), a focus on attachment and on enhancing adult-child relationships in a more general sense (six articles), and a focus on developmental considerations (eight articles). Four of the included articles described TICI that were implemented across pediatric and adult settings, without any reported adjustments made between settings (Craig & Sanders, 2018; Jacobowitz et al., 2015; McEvedy et al., 2017; Williams & Smith, 2017).

**Table 3.** TIC Intervention Aims.

Intervention Name	To Reduce Restraints/Seclusions/Critical Events	To Change Staff Attitudes, Practices	To Increase/Change Available Assessments & Treatments/Coordinate Care	To Support Staff Wellness/Increase Staff Self-Capacities	To Increase Pt Capacity/Improve Pt Outcomes	To Change Culture	Total AIM Themes Captured
Attachment, Regulation and Competency (ARC)	☑	☑	☑	☑	☑	☑	6
Collaborative Problem Solving (CPS)	☑	☑	☑	☑	☑	☑	6
Sensory Integration Initiatives	☑	☑	☑		☑	☑	5
Sanctuary Model			☑	☑	☑	☑	4
Trauma Affect Regulation: Guide for Education and Therapy (TARGET)	☑	☑			☑	☑	4
Children and Residential Experiences (CARE)		☑	☑			☑	3
EQ2: Empowering direct care staff to build trauma-responsive communities for youth		☑		☑		☑	3
NASMHPD six core strategies	☑	☑				☑	3
Trauma-Informed Psychiatric Residential Treatment (TI-PRT)	☑				☑	☑	3
Trauma-Systems Therapy (TST)		☑	☑		☑		3
Child Adult Relationship Enhancement (CARE)		☑	☑				2
Patient-Focused Intervention (PFI) Model	☑					☑	2
Structured Psychotherapy for Adolescents Responding to Chronic Stress (SPARCS)			☑		☑		2
Devereux’s Safe and Positive Approaches (SPA)		☑					1
Neurosequential Model of Therapeutics (NMT)						☑	1
Risking Connection (RC) and Restorative Approach (RA)						☑	1
Gender-specific and trauma-informed training curriculum		☑					1
Trauma-informed approach (TIA)	☑						1
Trauma-Informed Child Welfare Service (CWS)			☑				1
TIC Training Program			☑				1
A Trauma-Informed Care Program							0

### Implementation Strategies

#### Implementation goals

In Table 4 we present the list of implementation strategy goal categories along with a tabulation of the total TICI (n = 21) reporting the respective goals within their implementation strategies. Eleven interventions included five or more different implementation goal categories, while four articles (out of the included 49) reported only one implementation goal, and two articles reported no implementation strategy goals. The most commonly reported implementation goal was to increase knowledge and awareness (20 interventions), followed by changing behavior or practice (18 interventions) and informing or supporting implementation (17 interventions). In Table S2 we present the detailed implementation strategies and implementation goals by TICI (n = 21) including the corresponding ERIC categories and implementation goal categories. **Implementation strategy (ERIC) themes.** All 21 included TICI incorporated the implementation theme of training and educating stakeholders, and almost all (18 of the 21 interventions) included the implementation theme of developing stakeholder interrelationships. The remaining eight implementation strategy themes were used to implement with approximately half of the interventions. See Table S3 for a list of the ERIC implementation strategies contained within each overarching theme. Four of the TICI included implementation strategies across all the 10 themes (ARC, NASMHPD Six Core Strategies, PFI, and the grouped sensory integration initiatives). The majority of the TICI (n = 11) incorporated strategies related to six or more implementation themes, while five interventions incorporated strategies from three or fewer implementation themes (see Table 5). **Implementation strategies (ERIC).** Of the 73 original ERIC strategies and the four added strategies, one strategy, “Conduct educational meetings” was used to implement all 21 TICI, with two TICI only using this implementation strategy (see Table S4). In addition to educational meetings, there were seven other ERIC strategies used by more than half of the interventions: E27 “Develop and organize quality monitoring systems”, E65 “Use an implementation advisor”, E19 “Conduct ongoing training”, E55 “Provide ongoing consultation”, E71 “Use train-the-trainer strategies”, E41b “Involve staff”, and E44 “Mandate change”. Most TICI reported using more than 10 ERIC strategies, with four interventions (NASMHPD Six Core Strategies, ARC, the grouped sensory integration initiatives, and the Sanctuary Model) using more than 30 ERIC strategies. Sixteen ERIC implementation strategies were not used with any of the included TICI. Six of the nine strategies under the theme “H: Utilize financial strategies” and three of the eight strategies under the theme “I: Change infrastructure” were unused.

**Table 4.** Implementation Strategy Goal Categories by TIC Intervention (*n* = 21).

Goal of the Implementation Strategy
1. Increase knowledge/awareness (*n* = 20; 95%)
2. Inform/change attitudes (*n* = 3; 14%)
3. Inform/change behavior/practice (*n* = 18; 86%)
4. Inform/change policy (*n* = 5; 24%)
5. Increase patient/family involvement (*n* = 4; 19%)
6. Increase staff involvement (*n* = 7; 33%)
7. Inform/support implementation (*n* = 17; 81%)
8. Embed the model (*n* = 12; 57%)

**Table 5.** ERIC Overarching Themes Used With Each TIC Intervention.

Intervention Name	Total ERIC Themes	A. Use Evaluative and Iterative	B. Provide Interactive	C. Adapt and Tailor to Context	D. Develop Stakeholder	E. Train and Educate	F. Support Clinicians	G. Engage Consumers & Staff	H. Utilize Financial Strategies	I. Change Infrastructure	J. Not Categorized
NASMHPD Six Core Strategies	10	☑	☑	☑	☑	☑	☑	☑	☑	☑	☑
Attachment, Regulation and Competency (ARC)	10	☑	☑	☑	☑	☑	☑	☑	☑	☑	☑
Sensory integration initiatives	10	☑	☑	☑	☑	☑	☑	☑	☑	☑	☑
Patient-Focused Intervention (PFI) Model	10	☑	☑	☑	☑	☑	☑	☑	☑	☑	☑
Sanctuary Model	9	☑	☑	☑	☑	☑	☑	☑		☑	☑
Collaborative Problem Solving (CPS)	9	☑	☑		☑	☑	☑	☑	☑	☑	☑
Trauma-Systems Therapy (TST)	9	☑	☑	☑	☑	☑		☑	☑	☑	☑
Trauma-Informed Child Welfare Service (CWS)	8	☑			☑	☑	☑	☑	☑	☑	☑
Trauma-informed approach (TIA)	7	☑	☑			☑	☑	☑		☑	☑
EQ2: Empowering direct care staff to build trauma-responsive	6	☑		☑	☑	☑	☑	☑			
Trauma Affect Regulation: Guide for Education and Therapy (TARGET)	6		☑	☑	☑	☑		☑		☑	
Neuro sequential Model of Therapeutics (NMT)	5	☑		☑	☑	☑				☑	
Child Adult Relationship Enhancement (CARE)	5		☑		☑	☑		☑	☑		
Structured Psychotherapy for Adolescents Responding to Chronic	5		☑	☑	☑	☑	☑				
Trauma-Informed Psychiatric Residential Treatment (TI-PRT)	5	☑	☑		☑	☑		☑			
TIC Training Program	4	☑			☑	☑			☑		
Children and Residential Experiences (CARE)	3			☑	☑	☑					
Risking Connection (RC) and Restorative Approach (RA)	3				☑	☑				☑	
Gender-Specific and Trauma-Informed Training Curriculum	2				☑	☑					
Devereux’s Safe and Positive Approaches (SPA)	1					☑					
A Trauma-Informed Care Program	1					☑					
Total TICI using theme		13	12	11	18	21	10	13	9	12	9

## Discussion

### Recent Proliferation of TICI

In this scoping review, we identified 21 TICI in pediatric mental health inpatient and residential settings across 49 articles. While our database search spanned from 1995 onwards, the earliest article we identified was published in 2003 and most articles (n = 35) were published since 2010. The reasons for the recent proliferation of different TICI and approaches may be numerous. First, settings and populations are diverse and complex and require specialized adaptations according to the context in which the interventions are to be implemented. Second, and perhaps more importantly, TICI that are better known and more established are not widely accessible to the public; they tend to be quite expensive to purchase and implement and require a substantial commitment of time, effort, and resources (Hanson & Lang, 2016). For example, in addition to the cost of purchasing the Sanctuary Model, there may be further internal resources required, as the model is designed to be implemented over three years (Andrus, 2022). To obtain the certification, organizations must demonstrate that they have met all 28 of the Sanctuary Institute Standards for Certification (Sanctuary Institute, 2021). Organizations may therefore opt to design and develop their own less expensive and comprehensive approaches. Third, as demonstrated in this review, the reporting of TICI and their implementation is often limited and lacks detail, making them difficult to replicate. Fourth, various definitions of TIC exist (e.g., Bath, 2008; Becker-Blease, 2017; Hanson & Lang, 2016; SAMHSA, 2014; The National Child Traumatic Stress Institute, n.d.). We have yet to achieve a consensus on what TIC actually represents, and the elements and mechanisms needed to achieve it (Hanson & Lang, 2016; Perry, 2020). Hence, it logically follows that depending on the TIC definition adopted by organizations aiming to implement TIC, various TICI could be developed to achieve their desired goals.

### Complexity of TIC: TICI Aims, Targets, and Implementation

In this scoping review we identified TICI that endeavour to operationalize the complexity of TIC through various intervention aims, target audiences, and multifaceted interventions. In many cases, it was difficult to identify the TICI aims (distinct from the study aims). When explicitly reported, they varied, potentially indicating that the TICI were designed for different purposes, or possibly representing a continuum of the multiple levels along the complexity of TIC. Aims ranged from abstract to narrower and more specific. TICI specifically targeted patients (e.g., Clarke, 2012; Forrest et al., 2018; Habib, et al., 2013; Marrow et al., 2012), staff (e.g., Crable et al., 2013; Hale & Wendler, 2023; Russell et al., 2009), and caregivers (e.g., Hodgdon et al., 2013; Regan et al., 2017), or a combination thereof. While it may seem evident to target patients with TICI, some authors posit the importance of also including direct care staff (e.g., Wolf et al., 2014) and caregivers (e.g., Lotty et al., 2020; Sullivan et al., 2016) in the cultivation of trauma-informed organizations that engender safety, trustworthiness, support, collaboration, and empowerment. Moreover, perhaps we need to consider not only multiple target audiences but also the complexity of how these audiences interact dynamically within TICI. This may be especially true within the pediatric context, where caregivers play an essential role in supporting and bridging the youth to the community. Eight of the twenty-one TICI aimed to reduce seclusion and restraint events. Yet all those TICI also encompassed aims that went beyond and targeted other areas such as increasing patient capacities, changing staff attitudes, and bringing about a broader culture change, seeming to indicate a general consensus that TIC aims go well beyond simply reducing restraints and seclusions. The most common aim, stated by 12 of the 21 of TICI included in this review, was to promote a form of organizational culture change. While the higher-level aim of culture change is congruent with the complexity of TIC, we also need to consider how this can be operationalized in practice (Hanson & Lang, 2016). A recent example of a TICI (not included in this review, as it is not published in the peer-reviewed literature) that considers the larger cultural context and operationalization of TIC, is a tool developed by the National Children Traumatic Stress Network (NSTCN) called the Trauma-Informed Organizational Assessment (TIOA; Halladay Goldman et al., 2019). The TIOA assesses nine broad aspects of TIC within the organization (covering youth, caregiver, and staff domains) and includes a detailed implementation guide exhibiting the complexity of TIC and the complexity of implementing a TICI.

We also found a large degree of variability in the nature, scope, and number of implementation strategies used to implement each TICI, which is consistent with findings of other reviews of TICI (e.g., Bryson et al., 2017; Lowenthal, 2020). Some TICI used more than half of the 77 ERIC implementation strategies, while others used very few, but the majority did use a multipronged implementation plan. All the included TICI used educational meetings as an implementation strategy with two using educational meetings as the sole implementation strategy. Yet, educational meetings as an exclusive strategy are likely to fall short in achieving the commonly targeted culture change. This was illustrated by authors of two TICI included in this review. Williams and Smith (2017) reported that while training was effective in increasing knowledge and altering attitudes, training had less effect on changing individual practice and an even lower level of influence on changing workplace practice. Lang et al. (2016) also flagged the shortcomings of educational meetings in producing practice changes and warned that “as interest in trauma-informed care grows, there is a risk that ‘receiving some trauma-related training’ becomes equivalent to ‘being trauma-informed’” (Lang et al., 2016, pp 121-122).

Lowenthal (2020), in their review of TIC implementation in the child- and youth-serving sectors, created a three-category post-hoc analytic framework called the TIC Implementation Scope Continuum to classify the nature, scope, and number of implementation strategies of the range of TIC implementation initiatives. Limited Change Initiatives (LCI) consisted generally of one-off trainings with little to no follow up, while Comprehensive Change Initiatives (CCI) used numerous strategies over longer periods of time to create lasting changes in organizational culture, structure, and policies. While this framework offers potential to classify the nature and scope of TIC implementation initiatives, the framework needs to be studied to establish reliability and validity. Further, an important direction for future focus would be to investigate the association between their comprehensiveness and intensity and the effectiveness of the TICI and its implementation.

### Reporting of TICI and Implementation Strategies

In conducting this scoping review, we found the reporting of both the TICI and of the implementation strategies to be varied and limited. Some articles did not describe or described very minimally the intervention and/or the implementation strategies, so we suggest caution when reviewing and interpreting what was actually done. We note from these articles that there is confusion or lack of clarity around the TICI in contrast to strategies used to implement it. Some articles reported primarily the implementation strategies (such as describing the TICI as a “training”) rather than what the TICI was in practice. From an implementation perspective, a training would be an implementation strategy to facilitate the adoption of the core clinical intervention, whatever that may be (Eldh et al., 2017). To achieve greater clarity and consistency in the reporting of TICI will necessitate further elucidation and agreement around each of these concepts: TIC definitions, TICI, and TIC implementation strategies. Moreover, intervention frameworks or reporting guidelines should be adopted to aid in consistent reporting of and to distinguish between clinical and implementation interventions or strategies (Eldh et al., 2017). None of the included articles in this review incorporated intervention frameworks or reporting guidelines, which made the TICI difficult to synthesize. There are a number of intervention reporting guidelines in the EQUATOR (Enhancing the Quality and Transparency of Health Research network; Simera et al., 2010) database that may be useful to consider in identifying the most useful guidelines for the reporting of TICI, such as the Template for Intervention Description and Replication (TIDieR) checklist (Hoffmann et al., 2014), and the Standards for Reporting Implementation Studies (StaRI) checklist (Pinnock et al., 2017). In our scoping review protocol, we initially selected the (TIDieR) Checklist to guide our data extraction of the TICI and of the implementation strategies. After commencing data extraction, we realized TIDieR Checklist incorporated detailed elements that for the most part were not relevant to the reported TICI that we had identified. After reviewing other available frameworks, we opted to use the AIMD framework (Bragge et al., 2017) as a structure for breaking down and discussing the TICI. Furthermore, the Expert Recommendations for Implementing Change (ERIC; Powell et al., 2015) as a taxonomy for classifying the implementation strategies proved useful for classifying/categorizing strategies used to implement TICI. While intervention frameworks and reporting guidelines can structure and facilitate reporting, the onus remains on the authors to report completely and transparently what took place, otherwise we are still left with incomplete and inconsistent reporting. We encourage authors to enhance the descriptive clarity of the TICI and implementation strategies used with the support of available intervention frameworks and reporting guidelines. See Table 6 for a summary of the implications from this review findings for practice, policy, and research.

**Table 6.** Implications for Practice, Policy, and Research.

Implications for Practice • Numerous TIC interventions for pediatric settings exist • These TICI vary in their aims and essential components • Organizations looking to implement TICI need to consider their goals for TIC, which will inform who the TICI will target (e.g., patients, staff, caregivers) and TICI aims (e.g., reducing incidence of critical events, improving patient outcomes, changing culture) • In planning implementation of TICI, consider using a multi-pronged approach that goes beyond educational trainings (education is necessary but not sufficient to support implementation) • Given that the amount of detail reported on TICI and implementation strategies is limited, organizations may need to explore and create their own implementation plans based on the needs of their setting • Organizations should monitor and document the impact of their implementation efforts
Implications for Policy • When planning implementation of a TIC intervention, policy makers should clearly identify the specific goals that they want the TIC intervention to achieve • Policy makers have an important role in supporting the implementation of TIC interventions (as evidenced by many of the implementation strategies which have implications for policy providers - e.g. Using evaluative and iterative strategies, Developing knowledge user interrelationships, Supporting clinicians, and Changing infrastructure)
Implications for Research • Researchers need to do better in describing the TIC interventions and the implementation strategies used • More research is needed to better understand what combination of implementation strategies work best for what TIC interventions and for specific contexts

## Study Limitations

Our search strategy included the terms of “trauma-informed”, “trauma-sensitive”, “trauma-integrated”, and “trauma * care” which are commonly used to describe this phenomenon (e.g., Hanson & Lang, 2016), but it is possible additional relevant articles exist that were not indexed with these terms. Due to feasibility concerns, we excluded non-peer reviewed articles, including theses, as well as articles published in languages outsides of English or French; in doing so, we may have missed relevant TICI. It is also possible that others may have coded data differently (e.g., when applying the ERIC implementation strategies as categories). However, our citation screening, data extraction, and data coding processes involved two independent team members and reviewed with the larger team when consensus or clarification was needed leading us to believe that the chance of miscoding was small. We did not assess the quality of the included studies, however this is consistent with guidance related to conducting scoping reviews (Peters et al., 2020). This review focused on pediatric settings and therefore we could not compare how TICI within adult and pediatric settings differ, however this may be a valuable area for future research.

Most articles identified in this scoping review were based in the USA, with a few based in Canada and Australia, and one article including sites from Canada, Scotland, and USA. Given that the healthcare system in the USA differs from other systems in the world, there may be limitations to the transferability of these interventions to other locations. Furthermore, the lack of published interventions implemented elsewhere in the world, including in from countries in the Global South, limits our ability to conceptualize TIC and TIC implementation within diverse settings and contexts. Finally, our analysis was limited to what was reported in articles (both in terms of the TICI and the implementation strategies). Authors may not have fully reported what was done in either the clinical intervention or the implementation.

## Conclusions

In conducting this scoping review, we identified numerous admirable efforts to implement TICI in pediatric inpatient and residential mental health settings, demonstrating a broad interest in TIC. The included TICI encompassed some common aims and elements, however, there were also many differences. In selecting, implementing, or reporting on a TIC intervention, it will be important for organizations to consider their goals for TIC and to describe the aims and core components of the TIC intervention separate from the implementation strategies to be used. This specificity will better allow for synthesis and transferability of TICI. We suggest that a tailored implementation plan should multi-pronged approach that goes well beyond educational trainings. We also recommend that further research focuses on developing a better understanding of what combination of implementation strategies work best for what TICI under specific contexts.

## Supplemental Material

sj-docx-1-tva-10.1177_15248380231193444 – Supplemental material for Trauma-Informed Care Interventions Used in Pediatric Inpatient or Residential Treatment Mental Health Settings and Strategies to Implement Them: A Scoping Review Supplemental material, sj-docx-1-tva-10.1177_15248380231193444 for Trauma-Informed Care Interventions Used in Pediatric Inpatient or Residential Treatment Mental Health Settings and Strategies to Implement Them: A Scoping Review by Yehudis Stokes, Krystina B. Lewis, Andrea C. Tricco, Erin Hambrick, Jean Daniel Jacob, Melissa Demery Varin, Justine Gould, Dhiraj Aggarwal, Paula Cloutier, Catherine Landriault, Stephanie Greenham, Michelle Ward, Allison Kennedy, Jennifer Boggett, Roxanna Sheppard, David Murphy, Marjorie Robb, Hazen Gandy, Sonia Lavergne and Ian D. Graham in Trauma, Violence, & Abuse

sj-docx-2-tva-10.1177_15248380231193444 – Supplemental material for Trauma-Informed Care Interventions Used in Pediatric Inpatient or Residential Treatment Mental Health Settings and Strategies to Implement Them: A Scoping Review Supplemental material, sj-docx-2-tva-10.1177_15248380231193444 for Trauma-Informed Care Interventions Used in Pediatric Inpatient or Residential Treatment Mental Health Settings and Strategies to Implement Them: A Scoping Review by Yehudis Stokes, Krystina B. Lewis, Andrea C. Tricco, Erin Hambrick, Jean Daniel Jacob, Melissa Demery Varin, Justine Gould, Dhiraj Aggarwal, Paula Cloutier, Catherine Landriault, Stephanie Greenham, Michelle Ward, Allison Kennedy, Jennifer Boggett, Roxanna Sheppard, David Murphy, Marjorie Robb, Hazen Gandy, Sonia Lavergne and Ian D. Graham in Trauma, Violence, & Abuse

sj-docx-3-tva-10.1177_15248380231193444 – Supplemental material for Trauma-Informed Care Interventions Used in Pediatric Inpatient or Residential Treatment Mental Health Settings and Strategies to Implement Them: A Scoping Review Supplemental material, sj-docx-3-tva-10.1177_15248380231193444 for Trauma-Informed Care Interventions Used in Pediatric Inpatient or Residential Treatment Mental Health Settings and Strategies to Implement Them: A Scoping Review by Yehudis Stokes, Krystina B. Lewis, Andrea C. Tricco, Erin Hambrick, Jean Daniel Jacob, Melissa Demery Varin, Justine Gould, Dhiraj Aggarwal, Paula Cloutier, Catherine Landriault, Stephanie Greenham, Michelle Ward, Allison Kennedy, Jennifer Boggett, Roxanna Sheppard, David Murphy, Marjorie Robb, Hazen Gandy, Sonia Lavergne and Ian D. Graham in Trauma, Violence, & Abuse

## References

[bibr1-15248380231193444] Andrus. (2022). Sanctuary Institute: Steps to implementation. https://www.thesanctuaryinstitute.org/services/steps-to-implementation/

[bibr2-15248380231193444] Azeem M. W. Aujla A. Rammerth M. Binsfeld G. Jones R. B. (2011). Effectiveness of Six Core Strategies based on trauma informed care in reducing seclusions and restraints at a child and adolescent psychiatric hospital. Journal of Child and Adolescent Psychiatric Nursing, 24(1), 11–15. 10.1111/j.1744-6171.2010.00262.x 21272110

[bibr3-15248380231193444] Azeem M. W. Reddy B. Wudarsky M. Carabetta L. Gregory F. Sarofin M. (2015). Restraint reduction at a pediatric psychiatric hospital: A ten-year journey. Journal of Child and Adolescent Psychiatric Nursing, 28(4), 180–184. 10.1111/jcap.12127 26549698

[bibr4-15248380231193444] Bailey C. Klas A. Cox R. Bergmeier H. Avery J. Skouteris H. (2019). Systematic review of organisation-wide, trauma-informed care models in out-of-home care (OoHC) settings. Health & Social Care in the Community, 27(3), e10–e22. 10.1111/hsc.12621 30033666

[bibr5-15248380231193444] Baker C. N. Brown S. M. Wilcox P. Verlenden J. M. Black C. L. Grant B.-J. E. (2018). The implementation and effect of trauma-informed care within residential youth services in rural Canada: A mixed methods case study. Psychological Trauma, 10(6), 666–674. 10.1037/tra0000327 29016153

[bibr6-15248380231193444] Barnett E. R. Yackley C. R. Licht E. S. (2018). Developing, implementing, and evaluating a trauma-informed care program within a youth residential treatment center and special needs school. Residential Treatment for Children & Youth, 35(2), 95–113. 10.1080/0886571X.2018.1455559

[bibr7-15248380231193444] Bath H. (2008). The three pillars of trauma-informed care. Reclaiming Children and Youth, 17(3), 17–21.

[bibr8-15248380231193444] Becker-Blease K. A. (2017). As the world becomes trauma-informed, work to do. Journal of Trauma & Dissociation, 18(2), 131–138. 10.1080/15299732.2017.1253401 28145820

[bibr9-15248380231193444] Bloom S. L. (2003). The Sanctuary Model: A trauma-informed systems approach to the residential treatment of children. Residential Group Care Quarterly, 4(2), 1–4.

[bibr10-15248380231193444] Bloom S. L. Bennington-Davis M. Farragher B. McCorkle D. Nice-Martini K. Wellbank K. (2003). Multiple opportunities for creating sanctuary. Psychiatric Quarterly, 74(2), 173–190. 10.1023/A:1021359828022 12602832

[bibr11-15248380231193444] Boel-Studt S. M. (2017). A quasi-experimental study of Trauma-Informed Psychiatric Residential Treatment for children and adolescents. Research on Social Work Practice, 27(3), 273–282. 10.1177/1049731515614401

[bibr12-15248380231193444] Bragge P. Grimshaw J. M. Lokker C. Colquhoun H. (2017). AIMD – a validated, simplified framework of interventions to promote and integrate evidence into health practices, systems, and policies. BMC Medical Research Methodology, 17(1), 38–38. 10.1186/s12874-017-0314-8 28259155 PMC5336675

[bibr13-15248380231193444] Brend D. Fréchette N. Milord-Nadon A. Harbinson T. Colin-Vezina D. (2020). Implementing trauma-informed care through social innovation in residential care facilities serving elementary school children. International Journal of Child and Adolescent Resilience, 7(1), 222–232. 10.7202/1072600ar

[bibr14-15248380231193444] Brown A. D. McCauley K. Navalta C. P. Saxe G. N. (2013). Trauma Systems Therapy in residential settings: Improving emotion regulation and the social environment of traumatized children and youth in congregate care. Journal of Family Violence, 28(7), 693–703. 10.1007/s10896-013-9542-9 24078769 PMC3782637

[bibr15-15248380231193444] Brown S. M. Baker C. N. Wilcox P. (2012). Risking connection trauma training: A pathway toward trauma-informed care in child congregate care settings. Psychological Trauma, 4(5), 507–515. 10.1037/a0025269

[bibr16-15248380231193444] Bryson S. Gauvin E. Jamieson A. Rathgeber M. Faulkner-Gibson L. Bell S. Davidson J. Russel J. Burke S. (2017). What are effective strategies for implementing trauma-informed care in youth inpatient psychiatric and residential treatment settings? A realist systematic review. International Journal of Mental Health Systems, 11(1), 36. 28503194 10.1186/s13033-017-0137-3 PMC5425975

[bibr17-15248380231193444] Caldwell B. Albert C. Azeem M. W. Beck S. Cocoros D. Cocoros T. Montes R. Reddy B. (2014). Successful seclusion and restraint prevention effort in child and adolescent programs. Journal of Psychosocial Nursing and Mental Health Services, 52(11), 30–38. 10.3928/02793695-20140922-01 25250792

[bibr18-15248380231193444] Canadian Institutes of Health Research. (2012). Guide to knowledge translation planning at CIHR: Integrated and end-of-grant approaches. https://cihr-irsc.gc.ca/e/documents/kt_lm_ktplan-en.pdf

[bibr19-15248380231193444] Chu J. A. (2011). Rebuilding shattered lives: Treating complex PTSD and dissociative disorders. John Wiley & Sons, Inc.

[bibr20-15248380231193444] Clarke A. (2012). Why the Sanctuary Model? Developing Practice, 31, 53–61

[bibr21-15248380231193444] Crable A. R. Underwood L. A. Parks-Savage A. Maclin V. (2013). An examination of a gender-specific and trauma-informed training curriculum: Implications for providers. International Journal of Behavioral and Consultation Therapy, 7(4), 30–37. 10.1037/h0100964

[bibr22-15248380231193444] Craig J. H. Sanders K. L. (2018). Evaluation of a program model for minimizing restraint and seclusion. Advances in Neurodevelopmental Disorders, 2(4), 344–352. 10.1007/s41252-018-0076-2

[bibr23-15248380231193444] Cuijpers P. Smit F. Unger F. Stikkelbroek Y. Ten Have M. De Graaf R. (2011). The disease burden of childhood adversities in adults: A population-based study. Child Abuse and Neglect, 35(11), 937–945. http://doi.org/10.1016/j.chiabu.2011.06.005 22099144 10.1016/j.chiabu.2011.06.005

[bibr24-15248380231193444] Denison M. Gerney A. Barbuti Van Leuken J. Conklin J. (2018). The attitudes and knowledge of residential treatment center staff members working with adolescents who have experienced trauma. Residential Treatment for Children & Youth, 35(2), 114–138. 10.1080/0886571X.2018.1458689

[bibr25-15248380231193444] Eldh A. C. Almost J. DeCorby-Watson K. Gifford W. Harvey G. Hasson H. Kenny D. Moodie S. Wallin L. Yost J. (2017). Clinical interventions, implementation interventions, and the potential greyness in between -a discussion paper. BMC Health Services Research, 17(1), 16. 10.1186/s12913-016-1958-5 28061856 PMC5219812

[bibr26-15248380231193444] Elliott D. E. Bjelajac P. Fallot R. D. Markoff L. S. Reed B. G. (2005). Trauma-informed or trauma-denied: Principles and implementation of trauma-informed services for women. Journal of Community Psychology, 33(4), 461–477. http://doi.org/10.1002/jcop.20063

[bibr27-15248380231193444] Esaki N. Hopson L. M. Middleton J. S. (2014). Sanctuary Model implementation from the perspective of indirect care staff. Families in Society, 95(4), 261–268. 10.1606/1044-3894.2014.95.31

[bibr28-15248380231193444] Fallot R. D. Harris M. (2009). Creating cultures of trauma-informed care (CCTIC): A self-assessment and planning protocol. Community Connections.

[bibr29-15248380231193444] Farragher B. Yanosy S. (2005) Creating a trauma-sensitive culture in residential treatment. Therapeutic Community, 26(1), 97–113.

[bibr30-15248380231193444] Felitti V. J. (2009). Adverse childhood experiences and adult health. Academic Pediatrics, 9(3), 131–132. http://doi.org/10.1016/j.acap.2009.03.001 19450768 10.1016/j.acap.2009.03.001

[bibr31-15248380231193444] Felitti V. J. Anda R. F. Nordenberg D. Williamson D. F. Spitz A. M. Edwards V. Koss M. P. Marks J. S. (1998). Relationship of childhood abuse and household dysfunction to many of the leading causes of death in adults: The adverse childhood experiences (ACE) study. American Journal of Preventive Medicine, 14(4), 245–258. http://doi.org/10.1016/S0749-3797(98)00017-8 9635069 10.1016/s0749-3797(98)00017-8

[bibr32-15248380231193444] Forrest S. Gervais R. Lord K. A. Sposato A. Martin L. Beserra K. Spinazzola J. (2018). Building communities of care: A comprehensive model for trauma-informed youth capacity building and behavior management in residential services. Residential Treatment for Children & Youth, 35(4), 265–285. 10.1080/0886571X.2018.1497930

[bibr33-15248380231193444] Goetz S. B. Taylor-Trujillo A. (2012). A change in culture: Violence prevention in an acute behavioral health setting. Journal of the American Psychiatric Nurses Association, 18(2), 96–103. 10.1177/1078390312439469 22442017

[bibr34-15248380231193444] Greene R. W. Ablon J. S. Martin A. (2006). Use of collaborative problem solving to reduce seclusion and restraint in child and adolescent inpatient units. Psychiatric Services, 57(5), 610–612. 16675751 10.1176/ps.2006.57.5.610

[bibr35-15248380231193444] Griffing S. Casarjian B. Maxim K. (2021). EQ2: Empowering direct care staff to build trauma-informed communities for youth. Residential Treatment for Children & Youth, 38(4), 362–380. 10.1080/0886571X.2020.1751018

[bibr36-15248380231193444] Gurwitch R. H. Messer E. P. Masse J. Olafson E. Boat B. W. Putnam F. W. (2016). Child–Adult Relationship Enhancement (CARE): An evidence-informed program for children with a history of trauma and other behavioral challenges. Child Abuse & Neglect, 53, 138–145. 10.1016/j.chiabu.2015.10.016 26613674

[bibr37-15248380231193444] Habib M. Labruna V. Newman J. (2013). Complex histories and complex presentations: Implementation of a manually-guided group treatment for traumatized adolescents. Journal of Family Violence, 28(7), 717–728. 10.1007/s10896-013-9532-y

[bibr38-15248380231193444] Hale R. Wendler M. C. (2023). Evidence-based practice: Implementing trauma-informed care of children and adolescents in the inpatient psychiatric setting. Journal of the American Psychiatric Nurses Association, 29(2), 161–170. 10.1177/1078390320980045 33349098

[bibr39-15248380231193444] Halladay Goldman J. Purbeck Trunzo C. Agosti J . (2019). NCTSN trauma-informed organizational assessment. National Center for Child Traumatic Stress. 10.1177/10775595251399911 41275411

[bibr40-15248380231193444] Hambrick E. P. Brawner T. W. Perry B. D. Wang E. Y. Griffin G. DeMarco T. Capparelli C. Grove T. Maikoetter M. O’Malley D. Paxton D. Freedle L. Friedman J. Mackenzie J. Perry K. M. Cudney P. Hartman J. Kuh E. Morris J. , . . . Strother M. (2018). Restraint and critical incident reduction following introduction of the Neurosequential Model of Therapeutics (NMT). Residential Treatment for Children & Youth, 35(1), 2–23. 10.1080/0886571X.2018.1425651

[bibr41-15248380231193444] Hanson R. Lang J. (2016). A critical look at trauma-informed care among agencies and systems serving maltreated youth and their families. Child Maltreatment, 21(2), 95–100. 10.1177/1077559516635274 26951344

[bibr42-15248380231193444] Harris M. Fallot R. D. (2001). Envisioning a trauma-informed service system: A vital paradigm shift. New Directions for Mental Health Services, 2001(89), 3–22. 10.1002/yd.23320018903 11291260

[bibr43-15248380231193444] Harris P.A. Taylor R. Thielke R. Payne J. Gonzalez N. Conde J. G. (2009). Research electronic data capture (REDCap)—a metadata-driven methodology and workflow process for providing translational research informatics support. Journal of Biomedical Informatics, 42(2), 377–381. 10.1016/j.jbi.2008.08.010 18929686 PMC2700030

[bibr44-15248380231193444] Hodgdon H. B. Kinniburgh K. Gabowitz D. Blaustein M. E. Spinazzola J. (2013). Development and implementation of trauma-informed programming in youth residential treatment centers using the ARC framework. Journal of Family Violence, 28(7), 679–692. 10.1007/s10896-013-9531-z

[bibr45-15248380231193444] Hoffmann T. C. Glasziou P. P. Boutron I. Milne R. Perera R. Moher D. Altman D. G. Barbour V. Macdonald H. Johnston M. Lamb S. E. Dixon-Woods M. McCulloch P. Wyatt J. C. Chan A. W. Michie S. (2014). Better reporting of interventions: Template for intervention description and replication (TIDieR) checklist and guide. BMJ, 358, g1687. 10.1136/bmj.g1687 pmid:24609605 24609605

[bibr46-15248380231193444] Huffhines L. Noser A. Patton S. R. (2016). The link between adverse childhood experiences and diabetes. Current Diabetes Reports, 16(6), 1–9. http://doi.org/10.1007/s11892-016-0740-8 27112958 10.1007/s11892-016-0740-8 PMC5292871

[bibr47-15248380231193444] Izzo C. V. Smith E. G. Holden M. J. Norton C. I. Nunno M. A. Sellers D. E. (2016). Intervening at the setting level to prevent behavioral incidents in residential child care: Efficacy of the CARE Program Model. Prevention Science, 17(5), 554–564. 10.1007/s11121-016-0649-0 27138932 PMC4887550

[bibr48-15248380231193444] Jacobowitz W. Moran C. Best C. Mensah L. (2015). Post-traumatic stress, trauma-informed care, and compassion fatigue in psychiatric hospital staff: A correlational study. Issues in Mental Health Nursing, 36(11), 890–899. 10.3109/01612840.2015.1055020 26631861

[bibr49-15248380231193444] Keeshin B. Strawn J. Luebbe A. Saldaña S. Wehry A. Delbello M. Putnam F. (2014). Hospitalized youth and child abuse: A systematic examination of psychiatric morbidity and clinical severity. Child Abuse & Neglect, 38(1), 76–83. 10.1016/j.chiabu.2013.08.013 24041456

[bibr50-15248380231193444] Lang J. M. Campbell K. Shanley P. Crusto C. A. Connell C. M. (2016). Building capacity for trauma-informed care in the child welfare system: Initial results of a statewide implementation. Child Maltreatment, 21(2), 113–124. 10.1177/1077559516635273 26928410

[bibr51-15248380231193444] LeBel J. Champagne T. (2010). Integrating sensory and trauma-informed interventions: A Massachusetts State Initiative, Part 2. Mental Health Special Interest Section Quarterly/American Occupational Therapy Association, 33(2), 1–4.

[bibr52-15248380231193444] LeBel J. Stromberg N. Duckworth K. Kerzner J. Goldstein R. Weeks M. Harper G. Sudders M. (2004). Child and adolescent inpatient restraint reduction: A state initiative to promote strength-based care. Journal of the American Academy of Child and Adolescent Psychiatry, 43(1), 37–45. 10.1097/00004583-200401000-00013 14691359

[bibr53-15248380231193444] Leigh-Smith C. Toth K. (2014). The Sanctuary Model, creating safety for an out-of-home care community. Children Australia, 39(4), 232–236. 10.1017/cha.2014.32

[bibr54-15248380231193444] Lotty M. Bantry-White E. Dunn-Galvin A. (2020). The experiences of foster carers and facilitators of Fostering Connections: The Trauma-informed Foster Care Program: A process study. Children and Youth Services Review, 119, 105516. 10.1016/j.childyouth.2020.105516

[bibr55-15248380231193444] Lowenthal A. (2020). Trauma-informed care implementation in the child- and youth-serving sectors: A scoping review. International Journal of Child and Adolescent Resilience, 7(1), 178–194. 10.7202/1072597ar

[bibr56-15248380231193444] Marrow M. T. Knudsen K. J. Olafson E. Bucher S. E. (2012). The value of implementing TARGET within a trauma-informed juvenile justice setting. Journal of Child & Adolescent Trauma, 5(3), 257–270. 10.1080/19361521.2012.697105

[bibr57-15248380231193444] Martin A. Krieg H. Esposito F. Stubbe D. Cardona L. (2008). Reduction of restraint and seclusion through Collaborative Problem Solving: A five-year prospective inpatient study. Psychiatric Services, 59(12), 1406–1412. 10.1176/ps.2008.59.12.1406 19033167

[bibr58-15248380231193444] Matte-Landry A. Collin-Vézina D. (2022). Patterns of change in restraints, seclusions and time-outs over the implementation of trauma-informed staff training programs in residential care for children and youth. Residential Treatment for Children & Youth, 39(2), 154–178. 10.1080/0886571X.2021.1929660

[bibr59-15248380231193444] McCorkle D. Peacock C. (2005). Trauma and the isms – a herd of elephants in the room: A training vignette. Therapeutic Communities, 26(1), 121–128.

[bibr60-15248380231193444] McEvedy S. Maguire T. Furness T. McKenna B. (2017). Sensory modulation and trauma- informed-care knowledge transfer and translation in mental health services in Victoria: Evaluation of a statewide train-the-trainer intervention. Nurse Education in Practice, 25, 36–42. 10.1016/j.nepr.2017.04.012 28477581

[bibr61-15248380231193444] McGowan S. Sampson M. Salzwedel D. M. Cogo E. Foerster V. Lefebvre C. (2016). PRESS peer review of electronic search strategies: 2015 guideline statement. Journal of Clinical Epidemiology, 75, 40–46. 10.1016/j.jclinepi.2016.01.021 27005575

[bibr62-15248380231193444] Murphy K. Moore K. A. Redd Z. Malm K. (2017). Trauma-informed child welfare systems and children’s well-being: A longitudinal evaluation of KVC’s bridging the way home initiative. Children and Youth Services Review, 75, 23–34. 10.1016/j.childyouth.2017.02.008

[bibr63-15248380231193444] Perry B. D. (2020). What is TIC? [Power Point slides]. Neurosequential Network.

[bibr64-15248380231193444] Perry C. K. Damschroder L. J. Hemler J. R. Woodson T. T. Ono S. S. Cohen D. J. (2019). Specifying and comparing implementation strategies across seven large implementation interventions: A practical application of theory. Implementation Science, 14(1), 32–32. 10.1186/s13012-019-0876-4 30898133 PMC6429753

[bibr65-15248380231193444] Peters M. D. J. Godfrey C. McInerney P. Munn Z Tricco A. C. Khalil H. (2020). Chapter 11: Scoping reviews. In Aromataris E. Munn Z. (Eds.), Joanna briggs institute reviewer’s manual. JBI. https://reviewersmanual.joannabriggs.org/

[bibr66-15248380231193444] Pinnock H. Barwick M. Carpenter C. R. Eldridge S. Grandes G. Griffiths C. J. Rycroft- Malone J. Meissner P. Murray E. Patel A. Sheikh A. Taylor S. J. C. (2017). Standards for Reporting Implementation Studies (StaRI) Statement. BMJ (Online), 356, i6795. 10.1136/bmj.i6795 PMC5421438 28264797

[bibr67-15248380231193444] Pollastri A. R. Lieberman R. E. Boldt S. L. Ablon J. S. (2016). Minimizing seclusion and restraint in youth residential and day treatment through site-wide implementation of Collaborative Problem Solving. Residential Treatment for Children & Youth, 33(3–4), 186–205. 10.1080/0886571X.2016.1188340

[bibr68-15248380231193444] Powell B. J. Waltz T. J. Chinman M. J. Damschroder L. J. Smith J. L. Matthieu M. M. Proctor E. K. Kirchner J. E. (2015). A refined compilation of implementation strategies: Results from the Expert Recommendations for Implementing Change (ERIC) project. Implementation Science, 10, 21. 25889199 10.1186/s13012-015-0209-1 PMC4328074

[bibr69-15248380231193444] Redd Z. Malm K. Moore K. Murphy K. Beltz M. (2017). KVC’s bridging the way home: An innovative approach to the application of Trauma Systems Therapy in child welfare. Children and Youth Services Review, 76, 170–180. 10.1016/j.childyouth.2017.02.013

[bibr70-15248380231193444] Regan K. (2010). Trauma informed care on an inpatient pediatric psychiatric unit and the emergence of ethical dilemmas as nurses evolved their practice. Issues in Mental Health Nursing, 31(3), 216–222. 10.3109/01612840903315841 20144033

[bibr71-15248380231193444] Regan K. M. Curtin C. Vorderer L. (2017). Paradigm shifts in inpatient psychiatric care of children: Approaching child- and family-centered care. Journal of Child and Adolescent Psychiatric Nursing, 30(4), 186–194. 10.1111/jcap.12193 30129238

[bibr72-15248380231193444] Rivard J. C. (2004). Initial findings of an evaluation of a trauma recovery framework in residential treatment. Residential Group Care Quarterly, 5(1), 3–5.

[bibr73-15248380231193444] Rivard J. C. Bloom S. L. Abramovitz R. Pasquale L. E. Duncan M. McCorkle D. Gelman A. (2003). Assessing the implementation and effects of a trauma-focused intervention for youths in residential treatment. Psychiatric Quarterly, 74(2), 137–154. 10.1023/A:1021355727114 12602830

[bibr74-15248380231193444] Rivard J. C. Bloom S. L. McCorkle D. Abramovitz R. (2005). Preliminary results of a study examining the implementation and effects of a trauma recovery framework for youths in residential treatment. Therapeutic Community, 26(1): 83–96.

[bibr75-15248380231193444] Rivard J. C. McCorkle D. Duncan M. E. Pasquale L. E. Bloom S. L. Abramovitz R. (2004). Implementing a trauma recovery framework for youths in residential treatment. Child & Adolescent Social Work Journal, 21(5), 529–550. 10.1023/B:CASW.0000043363.14978.e6

[bibr76-15248380231193444] Russell M. Maher C. Dorrell M. Pitcher C. Henderson L. (2009). A comparison between users and non-users of Devereux’s Safe and Positive Approaches Training Curricula in the reduction of injury and restraint. Residential Treatment for Children & Youth, 26(3), 209–220. 10.1080/08865710903130301

[bibr77-15248380231193444] Sanctuary Institute. (2021). Standards for certification. https://thesanctuaryinstitute.org/wp-content/uploads/2021/01/Standards-for-Certification-updated-January-2021.pdf

[bibr78-15248380231193444] Simera I. Moher D. Hoey J. Schulz K. F. Altman D. G. (2010). A catalogue of reporting guidelines for health research. European Journal of Clinical Investigation, 40(1), 35–53. 10.1111/j.1365-2362.2009.02234.x 20055895

[bibr79-15248380231193444] Stokes Y. Graham I. Lewis K. Tricco A. Jacob J.-D. Hambrick E. Paula Cloutier P. Aggarwal D. (2020). Identifying, implementing, measuring, and evaluating trauma- informed care interventions in pediatric mental health settings. osf.io/h9s4p

[bibr80-15248380231193444] Substance Abuse and Mental Health Services Administration (SAMHSA). (2014). SAMHSA’s concept of trauma and guidance for a trauma-informed approach. https://store.samhsa.gov/system/files/sma14-4884.pdf

[bibr81-15248380231193444] Sullivan K. M. Murray K. J. Ake G. S. (2016). Trauma-informed care for children in the child welfare system: An initial evaluation of a trauma-informed parenting workshop. Child Maltreatment, 21(2), 147–155. 10.1177/1077559515615961 26603357

[bibr82-15248380231193444] The National Child Traumatic Stress Institute (NCTSN). (n.d.). Creating trauma-informed systems. https://www.nctsn.org/trauma-informed-care/creating-trauma-informed-systems

[bibr83-15248380231193444] Tricco A. Lillie E. Zarin W. O’Brien K. Colquhoun H. Levac D. Moher D. Peters M. D. J. Horsley T. Weeks L. Hempel S. Akl E. A. Chang C. McGowan J. Stewart L. Hartling L. Aldcroft A. Wilson M. G. Garritty C. . . . Straus S. E. (2018). PRISMA extension for scoping reviews (PRISMA-ScR): Checklist and explanation. Annals of Internal Medicine, 169(7), 467–473. 10.7326/M18-0850 30178033

[bibr84-15248380231193444] Waltz T. J. Powell B. J. Matthieu M. M. Damschroder L. J. Chinman M. J. Smith J. L. Proctor E. K. Kirchner J. E. (2015). Use of concept mapping to characterize relationships among implementation strategies and assess their feasibility and importance: Results from the Expert Recommendations for Implementing Change (ERIC) study. Implementation Science, 10(1), 109–109. 10.1186/s13012-015-0295-0 26249843 PMC4527340

[bibr85-15248380231193444] Warner E. Koomar J. Lary B. Cook A. (2013). Can the body change the score? Application of sensory modulation principles in the treatment of traumatized adolescents in residential settings. Journal of Family Violence, 28(7), 729–738. 10.1007/s10896-013-9535-8

[bibr86-15248380231193444] Williams T. M. Smith G. P. (2017). Does training change practice? A survey of clinicians and managers one year after training in trauma-informed care. The Journal of Mental Health Training, Education, and Practice, 12(3), 188–198. 10.1108/JMHTEP-02-2016-0016

[bibr87-15248380231193444] Wolf M. R. Green S. A. Nochajski T. H. Mendel W. E. Kusmaul N. S. (2014). “We”re civil servants’: The status of trauma-informed care in the community. Journal of Social Service Research, 40(1), 111–120. 10.1080/01488376.2013.845131

[bibr88-15248380231193444] Yatchmenoff D. Sundborg S. Davis M. (2017). Implementing trauma-informed care: Recommendations on the process. Advances in Social Work, 18(1), 167–185. 10.18060/21311

