# Supplementary material for: Trauma-Informed Care Interventions Used in Pediatric Inpatient or Residential Treatment Mental Health Settings and Strategies to Implement Them: A Scoping Review
Source: Trauma Violence Abuse. 2023 Sep 11;25(3):1737–55. doi: 10.1177/15248380231193444 (PMC11155220; doi:10.1177/15248380231193444)
Supplement: sj-docx-2-tva-10.1177_15248380231193444 – Supplemental material for Trauma-Informed Care Interventions Used in Pediatric Inpatient or Residential Treatment Mental Health Settings and Strategies to Implement Them: A Scoping Review [file sj-docx-2-tva-10.1177_15248380231193444.docx]

**Supplementary File B**

Table S2.

*Implementation Strategies Grouped by TIC Intervention (n=21)*

*See Table S3 for the legend of the ERIC Categories and Table 4 for the legend of the Implementation Goal Categories*

| **Intervention Name** | **Author/Year** | **Implementation Strategies**^ERIC Categories^ | **Stated Goals of Implementation Strategies**^Goal Categories^ |
| --- | --- | --- | --- |
| **Attachment, Self-regulation, and Competency (ARC)** |  |  |  |
| Project Penguin informed by Attachment, Regulation and Competency (ARC) and Positive Behavioural Interventions and Supports (PBIS) | Brend/2020 | a) Multiple innovators converged to create Program Penguin^24,52,65^  b) Awareness training, additional training days^15^  c) On-site coaching^55^, case studies^15^, clinical supervision^53^, implementation committees^48,65^  d) Supplementary training and support activities for leaders^15^  e) Calming rooms^11^  f) Development of on-site expertise^57^  g) Adapting interventions to each site^41b,51^  h) Multi-site exchanges^7^  i) Using data to inform decision making^27,32,46b^  j) Social Innovation approach, scaling up the innovation^41b,61^ | a) To pool efforts to improve the lived reality and outcomes for children in residential treatment centres^7^  b) To increase staff understanding of complex trauma^1^  c) NR  d) “To transfer the knowledge base from the implementation team to key individuals within each site”^1^  e) NR  f) “To transfer knowledge from the implementation team to the residential treatment centres making the program more independently sustainable”^1,8^  g) To ensure the interventions were relevant to local needs while maintaining efficacy^7^.  h) “To allow settings to learn from each other about effective strategies and problems encountered throughout the province.”^1,7^  i) “Both to document implementation, and to assess intervention effects”^7^  j) “To achieve lasting transformation through methods that include people with lived experience of the challenge being addressed and allows for the process of change to be scaled up to respond to the breadth of the social problem of concern”^8^ |
| Building Communities of Care (BCC) | Forrest/2018 | a) Needs assessment^18,64^  b) Model workgroup^64^  c) Train the trainer training^15,22,65,71^  d) Constant presence of highly qualified trainers^27^  e) Trainer meetings^55^  f) Recertification^19^ | a) “To assess the degree to which our residential schools, group homes and treatment centers were trauma-informed”^7^  b) “To develop an evidence-informed care model that integrated trauma-informed principles and behavior management into an organizational framework”^7^  c)NR  d)To ensure fidelity^7^  e) To monitor application of the model^7^  f) NR |
| ARC; Grow Strong/Stepping Stones | Hodgdon/2013 | a) Funding from SAMHSA to adapt and implement the ARC framework in residential settings^1^  b) Trauma-informed needs assessment^4,18,41ab^  c) Implementation teams: “Trauma teams”^48,65^  d) Training program staff^15,43,55,65^  e) Implementing milieu behavioral enhancement initiatives^27,29,31,32,33,41b,,58^  f) Implementation of individual and group treatment^15,27,29,31,55,,65^  g) Evaluating outcomes^27,67^  h) Sustaining trauma informed services:  i) trauma team leadership and focus^48,57^;  ii) policy and procedure modifications^74^; iii) orientation and ongoing training (including train the trainer)^19,71^ and iv) on-going evaluation^27^ | a)”To adapt and implement the ARC framework…”^3,7^  b)”To identify specific residential programs that demonstrated interest in and commitment to implementing trauma-informed programming”^7^ “assessing needs from the perspective of clients, families (when possible), and program staff”^5,6,7^  c) To develop and carry out “interventions that specifically targeted building a trauma-informed milieu”^3,7^  d)“To address educational needs as well as the need for all program staff to utilize a shared lens to understand client presentations and to select appropriate intervention strategies.”^1,3^“To increase [staff] attunement”^3^  e) To increase “caregiver affect management skills”^3^ and to decrease “vicarious trauma”^9^ ; “To build a culture that accepted discussion about caregivers’ emotional responses to clients.”^8^“To increase consistent responses to problematic risk behaviors as well as positive behaviors”^3^  f) To sustain the program^8^  g)NR  h) i)To sustain the program^8^; ii) “To incorporate both the acknowledgement of the impact of trauma on their clients and the importance of providing trauma-focused treatment.”^4^ iii) “to ensure that the trauma training occurs for both new staff members and on an ongoing basis”^8^ |
| Trauma-Informed Care (TIC) Training informed by the Substance Abuse and Mental Health Services Administration (SAMHSA) and Attachment, Regulation, Competency (ARC) | Matte-Landry/2021 | a) Training development^17,18,29,63^  b) Phase 1: Initial training^15,43,44^  c) Phase 2: Coaching and supervision sessions^19,55^  d) Phase 3: Communities of practice, annual symposium, meetings with senior managers^7,15,41b,48,52,56^ | a) Training developed with stakeholders: “in order to ensure the acceptability, appropriateness, and feasibility of the proposed training”^7^  b) Training: “included teaching modules on SAMHSA principles and intervention targets from the ARC”^1^ “This part of the training thus targeted the workers’ skills and the units’ policies and intervention plans”^3,4^  c) To complete case vignettes and create action plans^7^; To allow “for in-depth discussions of alternatives of restrictive measures”^1^  d)Communities of practice: “Devoted to sharing experiences and resources and discuss global advancements in TIC knowledge, practices and policies”^1,7^ ; Yearly symposium: where renowned researchers presented information on TIC^1^; Regular meetings with upper management: “to address adoption and sustainability issues, such as high staff turnover”^7^ |
| **Child Adult Relationship Enhancement (CARE)** |  |  |  |
| Child Adult Relationship Enhancement (CARE) | Gurwitch/2016 | a) CARE training^15,31,33,41b,43,45^  b) Delaware Initiative: Funding^1^  c) Delaware Initiative: Training^15,31,55,65,71^ | a) “…are then taught the first components of CARE… to connect and engage with them [the child]”^1,3^  b)NR  c) Train the trainer: ““to teach the CARE model”^1,7^ |
| **Children and Residential Experiences (CARE)** |  |  |  |
| Children and Residential Experiences (CARE) | Izzo/2016 | a) Training and on-site consultation^15,19,55,65,71^  b) Local implementation determined by agency leaders and staff using creativity and professional judgment^51,63^  c) CARE Implementation Team (IT)^35,48^ | a) Train the trainer: “agency based trainers are prepared to deliver the same 5-day training to remaining staff”^1;^ Consultation: “supporting and facilitating day-to-day application of the principles in both childcare and staff management arenas”^3^  b) “cultivates personal investment and ownership and serves to reduce the sense of being constrained or controlled that is often elicited by more directive program models”^6^  c) “Its role involves providing support, modeling, and mentoring to staff as they incorporate CARE principles into their work. The team also builds structures and processes that facilitate application of the CARE principles and their eventual integration into the agency culture.”^3,8^ |
| **Collaborative Problem Solving (CPS)** |  |  |  |
| CPS | Greene/2006  Martin/2008 | a) Staff-wide training^15,31,65^  b) Supervision sessions^48,53^  c) Updating documentation systems^12^  d)Data collection^27,77^  e) Inclusion of family and caregivers in treatment^15,50^ | a)Schedule of training: To maximize exposure to the model for all staff members^1^  b) NR  c) “to maintain the CPS model incorporated into the unit’s clinical  practices after the active implementation  phase was over”^8^[Martin/2008]  d) NR  e) Family education: “to teach the basic elements of the model”^1^[Martin/2008] |
| CPS | Pollastri/2015 | Pre-implementation:  a) Identifying the need and implementation planning^15,40,44^  Active implementation:  b) Training (Intro, Tier 1, Tier 2, brief for admin and support staff)^15,65^  c) Phone/video coaching and supervision^53,55^  d) Certification for internal staff to provide training^71^  e) Reviews of restrictive interventions^32^  f) Language adjusted in clinical case presentations and clinical documentation^74^  Maintenance:  g) Ongoing training provided mostly by internal trainers^2,19,22,59,71^  h) Internal coaching provided by most highly trained staff.^19^  i) Monthly maintenance coaching^55^  j) Leaders talked with staff and managers^33,56^  k) Funding from community trainings^1^ | a) To identify an approach that would provide adequate “guidance in how to respond in trauma-informed ways to situations in which youth were not meeting adult expectations.”^1^  b) NR c) Tier 2 Supervision: “to discuss integrating the model into clinical programming and documentation.”^3,7^  d) NR  e) NR  f) NR  g) NR  h)NR  i) “To address challenges with CPS fidelity at the provider or organizational level”^7^  j) To inform and support implementation^7^  k) NR |
| Child and Family Centered Care (CFCC): CPS, Open Hours, and Trauma-Sensitive Protocols | Regan/2010  Regan/2017 | a) Trainings and supervision^15,53^  b) Focus groups with youth and consensus with staff^17,41b,46a^  c) Code of ethics poster^58^  d) Staffing switches upon request^41b,74^ | a) Training: To disseminate information “to staff regarding the dangers involved in physical restraints of children and adults”^1^[Regan/2010]  b)NR  c)NR  d)NR |
| **Devereux’s Safe and Positive Approaches (SPA)** |  |  |  |
| SPA | Russell/2009 | a) Training and supervisory training^15^ | a) To provide “strategies of preventing and limiting crisis situations, methods of intervention using a non-physical approach”^1^; To present “safe and effective physical intervention techniques”^1,3^ |
| **EQ2: Empowering Direct Care Staff to Build Trauma-Responsive Communities for Youth** |  |  |  |
| EQ2: Empowering Direct Care Staff to Build Trauma-Responsive Communities for Youth | Griffing/2020 | a) Planning meeting prior to implementation^4,23^  b)The “Circle” sessions^15,31,41b,43,48,51^  c) Circle booster sessions^19^  d) Weekly follow up contacts^46b,56,63^  e) “Checklists tracking completed components within each session”^27^  f) “An application (app)”^58^  g) An online e-learning program using a train-the  trainer model, in development^29,43,71^  h) Tracking of recruitment and retention rates and weekly attendance^27,56^  i) Follow-up interviews with program directors^56^  j) EQ2 Survey^46b,56^ | a) “To: i) determine the most effective way to introduce EQ2 to program staff; ii) discuss the optional program evaluation component; iii) review programmatic needs and potential implementation barriers; and, iv) plan logistics, with attention to the challenges in delivering services in residential programs that require continuous staff supervision”^7^  b) To strengthen “community engagement and collaboratively identifying nonpunitive approaches to managing challenging behaviors.”^1.6^  “To promote staff voice through the sharing of personal experiences,  which normalizes the stressors inherent in direct-care work.”^2,6^  c) “To help reinforce the concepts and skills presented during core EQ2 groups”^1,3^  d) “To obtain feedback and address any logistical and/or  clinical issues.”^7^ “To discuss possible adaptations over the course of the intervention.”^7^  e) “ To increase implementation fidelity across sites” (of the Circle sessions)^7^  f) “To reinforce the use of these self-regulation and mindfulness skills”^3^  g) NR  h) To assess “feasibility”^7^  i) To assess “feasibility”^7^  j) To assess “acceptability and efficacy” of the intervention^7^ |
| **NASMHPD Six Core Strategies** |  |  |  |
| NASMHPD Six Core Strategies | Azeem/2011 | a) Leadership towards organizational change^2,15,19,23,27,40,44,48^  b) Use of data to inform practice^5,27,32,33^  c) Workforce development^15,19,58,59^  d) Use of restraint and seclusion reduction tools^15,32^  e) Improve consumer’s role^41ab,46ab,50^  f) Debriefing techniques^27,32,33,41ab^ | a) To bring “major culture change”^8^, “to explain and inform staff”^1^, “to look at the progress and to implement various strategies [Trauma Reduction Team]”^7^  b) “Clinical reviews were conducted… to look at various reasons for seclusions and restraints and what can be done in the future to prevent them.”^7^  c)To create a “treatment environment that was based on Trauma Informed Care, facilitates recovery, and was inclusive”^8^  d)NR  e)NR  f) To identify “what went wrong, what could have been done differently, and how to avoid similar incidents in the future.”^7^; To implement “Interventions to mitigate the impact of traumatization and re-traumatization”^3^ |
| NASMHPD Six Core Strategies (and Building Bridges Initiative; BBI) | Azeem/2015  Caldwell/2014 | a) Leadership toward organizational change  [Solnit]^8, 39b,40,44,74^ [YDI]^40,44^ [MBA]^40,44^  b) Youth, family, and advocate involvement [Solnit]^2,13,41ab,46a,50,64^[YDI]^19,41ab,43,64^  c)Workforce development/Staff involvement [Solnit]^6,15,19,21,33,41ab,43,46b,55,72^  [YDI]^15,53^  [MPA]^6,15,41b,44,48^  d)Prevention tools [Solnit]^13,33,35,41ab,51,54,74^  [YDI :Caldwell]^11,41ab,44,58^  e)Debriefing [Solnit:Azeem/Caldwell]^33,39ab,41ab,46a,74^  f)Using data to inform practice [Solnit]^5,27,32,33,39b^  [MPA:Caldwell]^27,46a,57^  g) Environmental modifications: The healing bench [Solnit:Azeem, Caldwell]^11,58^ | a)[Solnit:Azeem/2015]: “to assist in operationalizing the tenets of optimal care, and for the provision of support and consultation for complex situations”^3^; [Solnit:Caldwell/2014]“to eliminate mechanical restraints”^3^;  [YDI; MPA]: NR  b)[Solnit:Azeem/2015]: “To build closer [family] relationships with staff at the hospital”^5^; “To identify areas of service and care in need of improvement”^7^; [Solnit:Caldwell/2014]:“To implement Solnit’s mission statement (i.e., “Caring, Healing and Teaching—Partnering with Children, Families and Communities to Build Hope and Create Opportunities)”^3^; “to involve both youth and families to better direct individualized care and service provision”^5^  [YDI:Caldwell/2014]: “To develop a Student Advisory Board”^5^  c) [Solnit:Azeem/2015] “To keep focus on the health of staff a priority”^6^  [Solnit:Caldwell/2014] To “shift towards a treatment culture change”^8^ Staff surveys: “Staff surveys were used to monitor staff satisfaction and training needs”^7^  [YDI:Caldwell/2014] “To support (a) the integration of new skills into staff’s work with youths and (b) staff’s ability to teach the same skills to families.”^3^  [MPA:Caldwell/2014]: Weekly meetings: “to address and eliminate any barriers that may impact success in making agency goals and values a reality.”^7^ “To getting all staff to join together to become educated on the new principles and practices”^1,3^  d) [Solnit: Azeem] “To intervene and support youth with unsafe behaviours”^3^  “to begin the process of engagement and ease their transition into the hospital program”^3,5^ “Staff have worked with family members to identify strategies [and recreational activities] that work for de-escalation of youth.”^3,5^  [YDI:Caldwell/2014] To eliminate the use of restraint.^3^  e) [Solnit:Azeem]: To prevent future restraint episodes^3^  [YDI:Caldwell/2014]: The goal of this [debriefing] approach is to resolve the conflict that has occurred and ensure planning to repair the relationship.^3^  f)[Solnit:Azeem/2015]:To build strategies to pre-empt future occurrences of restraint*s*^3,7^  [Solnit: Caldwell/2014]: NR  [MPA:Caldwell/2014]:“To help inform needed improvements.”^7^  g) [Solnit:Caldwell/2014]: To remind staff, children, and families of the work that has been done to reduce restraint use^1^ |
|  |  |  |  |
| ‘Broad TIC program’ based on Six Core Strategies and Risking Connection (RC) | Barnett/2018 | a) Administration Buy-in and Planning^15,17,24,40,41a,65^  b) Initial needs assessment^4,18,41b^  c) Trauma trainings and sustainment^15,16,19,53,55,57,71^  d) Internal sustainment of the program (incentives)^2^ | a) “To speak with the leadership board and board of directors about trauma and TIC as it applied to their programs”^1^ ”To obtain leadership board interest in and approval of the project”^7^  b) “To understand the needs of the facility and assist in planning the intervention program.”^1,7^  c) Train the Trainer Model: “To sustain the activities”^1,8^  d) “As an incentive for staff to participate in the program”^3^ |
| TIC Program based on the NASMHPD Six Core Strategies | Hale/2020 | a)Leadership commitment to organizational change^21,27,35,40,41ab,44,48,77^  b)Workforce development: Staff education^15^, all-star program^2^, and debriefings^33,41ab,46a,74^  c) Administrative updates: policies^44,^ job descriptions^59^, employee award^2^, patient documentation^12^  d)Enhanced communication: multidisciplinary discussions^74^  e) Use of data to inform the practice change^2,5,27^ | a) To review “data and effectiveness of interventions as they rolled out” [the Restraint and Seclusion Performance Improvement Team]^7^  b) To educate in the TIC philosophy and expected outcomes^1,^ Debriefings: “As a learning tool”^1^, To evaluate the use of de-escalation techniques and to teach alternatives^1,3,7^  c) “Policies and procedures related to the use of physical holds and seclusions were updated to reflect the processes and expected outcomes of infusion of TIC philosophy”^4,8^ “Job descriptions were updated to include requirements to use TIC”^3^, to recognize “excellent employee use of TIC actions”^3^, documentation updated “to uphold the principles and practice of TIC”^3^  d) To use “individual patient data … to inform practice”^3^  e) “Data… was shared with staff and patients, and used to evaluate adoption of the intervention of each floor”^7^ |
| **Neurosequential Model of Therapeutics**  **(NMT)** |  |  |  |
| NMT | Hambrick/2018 | a) Training and capacity building^7,15,19,22,23, 31,43,51,65,71^  b) Bi-annual fidelity assessments^26^ | a) “For individual clinicians and organizations/ sites to learn and implement [and be certified] this approach”^1,7^  b) To monitor and evaluate the clinician and site fidelity^7^ |
| **Patient-Focused Intervention (PFI) Model** |  |  |  |
| PFI | Barnum Goetz/2012 | a) Leadership involvement^40,44, 57, 63, 65^  b) Education^15, 16,74^  c) Regular staff certification^19^  d) Train-the-trainer model^71^  e) Code event review^5, 27,32, 33, 39b, 41b^  f) Quality feedback^5,27,32,63,^  g) Collaboration and consultation^6,41ab,46ab, 56, 63,65,^  h) Shared governance^32,41b,46b,52,64^  i) Celebrations^2^  j) Plan-do-check-act framework^14^ | a) “To improve and refine the model”^7^  b) “To introduce trauma-free concepts” ^1^, “introduce the local staff and community providers to these principles” ^1^, “To help reduce the violence potential of the patients.”, to manage aggression^1,3,4^  c)Not reported  d) “To assist with the adoption of the SWA [Sorensen and Wilder Associates aggression management program] process” ^1,3^, “increase trust in the SWA process”^2^, “act as role models for other staff” ^8^  e) “To reinforce education of the model and identify areas for improvement”^1,7^  f) “To determine areas of success and opportunities for improvement in the staff interventions being implemented”^3,7^, “To increase the involvement of the medical staff and administration in the process”^6^, “To both inform the leadership team and raise awareness of the outcomes of the project for the entire staff”^1^  g) “To improve the ongoing processes at the hospital” ^4^, “To identify improvements needed in the psychiatric inpatient programming.”^7^  h) “To make recommendations for changes in current practices, policies, and procedures and the current model of care”^3,4,7,8^  i) “To accentuate the demonstrated successes”^3^  j) “To manage the change” based on a theory^7^ |
| **Risking Connection (RC) and Restorative Approach (RA)** |  |  |  |
| RC and RA | Baker/2018 | a) Leadership consultation^55,65^  b) Training: Basic RC training, RA training^15,16,44,65^  c) Train the trainer^15,35,65,71^  d) Trainer and mentor guidance on the floor^60^ | a) “To shift the policies, procedures, and practices of the organization toward TIC”^3,4^  b)RC training: “To increase awareness about the prevalence and impact of trauma and to shift helper perspectives to be more trauma-informed”^1^  RA training: To provide helpers with tools that they can use in their work with clients^3^  c) “To gain the internal capacity to conduct ongoing formal RC trainings using their own credentialed trainers”^8^ ; “To impart the content and skills necessary to formally and informally teach RC within an organization”^7^  d) To “model the approach ‘on the floor’, sustaining and further embedding RC and RA into the work environment”^8^ |
| RC and RA | Brown/2012 | a) RC Training^15,16,43,65,71^  b) Identifying trainers[Agencies B,C,D]^35,65^  c) Consultation and additional training [Agencies C &D]^15,55^ | a) “Aimed at creating a common language among a variety of agency professionals serving traumatized individuals”^1^  b)“To affect the greatest impact on the agency system”^8^  c)NR |
| **Sanctuary Model** |  |  |  |
| Sanctuary Model | Bloom/2003a  Bloom/2003b  Farragher/2005  McCorkle/2005 | Julia Dyckman Andrus Memorial Center:  a)Integration: Formation of a core team^6,15,29,35,48,65^;  b) Understanding trauma & Avoiding reenactment: Staff training^15,52^, meetings^44,48^, supervision^53,74^; Revamping assessment process^12^, Retreats with a multidisciplinary team^48^  c) Fighting rigidity: Encouraging flexibility and creativity^51^, training in new therapy models^15^  d) Embracing non-violence: Red flag reviews; mandatory restraint reviews^27,32,33,39b,41b,44^  e) Preparing Sanctuary facilitation team as trainers^15,71^  Hawthorne-Cedar Knolls:  f)A multidisciplinary workgroup creating an organizational constitution^64^  g)Crafting mission statements for each unit^6,41ab^  h)Collaboration with an educational institution^24^  i)Ongoing training and consultation^19,55^  j) Trainings, supervision, management retreat^15,48,52,53^  CRR Group Home:  k)Training and supervision^15,16,53,65^ | a)The core team “assumed responsibility for training the entire staff”[Bloom/2003a]^1,8^  “Through the Core Team, representatives from each department  were able to take an honest look at their own departments’ strengths, shortcomings … and most importantly the assumptions which had been  driving their current behaviors and functioning”[Farragher/2005]^1,7^ “One of the tasks of the Core Team was to train the staff in trauma theory and to teach them a common language – the language of trauma.” [Farragher/2005]^1^  b) For staff “to have a basic understanding of how traumatic experiences disrupt normal coping and functioning in individuals and systems: to teach staff to ask “what happened” rather than “what is wrong with you?”” [Farragher/2005]^1,3^; “to help staff identify and break through their assumptions about each other and begin to build trusting, supportive relationships” [Farragher/2005]^2^; to create a “safer environment… with reduced reliance on physical restraint” [Farragher/2005]^3,8^  c) For staff “to know that it is safe to try new things, as long as they stay within the shared values and beliefs of the organization” [Farragher/2005]^3,6^  d) “To understand and reformulate a plan, any time there is an incident of violence on campus” [Farragher/2005]^3^; “to discuss alternative or proactive responses for the future…[and] to debrief the incident and revise the safety plan.” [Farragher/2005]^3,7^  e)“to prepare them to become trainers for the rest of the institution”[Bloom/2003b]^1,8^  f)”to be the organizing framework for the day-to-day functioning of the program” [Bloom/2003b]^7^  g)”as part of a democratic process reinforcing group ownership” [Bloom/2003b]^5,6^  h)NR  i)NR  j) “The purpose of holding joint trainings [with all disciplines] is to allow for multiple perspectives…[and] to learn from one another”^1,6^ [McCorkle/2005], Supervision: “To validate and support staff in balance of what needs improvement”^6,7^ [McCorkle/2005]  k)NR |
| Sanctuary Model | Clarke/2012  Leigh-Smith/2014 | a) Training^15,16,65^  b) The core team and implementation consultation^31,35,48,65^  c) Supervision^53^  d) Educating foster carers^50^  e) Posters and hiring practices^44,58^  f)Team meetings^41b^  g)Quantitative and qualitative data collection^27^ | a) “To commence this process”^7^ [Clarke/2012]; “to provide a safe environment, offer them support in recovery from their trauma and instill a belief that they  can create a different future for themselves”^3^ [Leigh-Smith/2014], “Being informed of trauma theory should serve as a preventative measure, or as ‘universal precaution’ to protect carers from experiencing vicarious trauma”^1,6^ [Leigh-Smith/2014]  b) To support the team through the change and implementation process^7^  c) “To help address safety, manage their emotions, deal with personal grief or loss, and finally set goals to move forward”^3,6^ [Leigh-Smith/2014]  d) “To identify, in themselves, when they are having this experience and when they might require additional support”^1,5^ [Leigh-Smith/2014]  e) Hiring practices: “helps to gauge if new employees understand the  concepts, and if they are already implementing them within their practice.”^8^ [Leigh-Smith/2014]  f)NR  g)NR |
| Sanctuary Model | Esaki/2014 | a) Initial 5-day training on the model for key leaders^15^  b) Formation of core team, and indirect care core team^35,48,55,65^  c)Certification process^22^  d)Staff survey^27^ | a) NR  b) To be primary change agents who work with colleagues to implement the model^7^  c)In the evaluation process the agency “is evaluated for successful implementation”^7^  d) “To assess the implementation of the Sanctuary Model”^7^ |
| Sanctuary Model | Rivard/2003  Rivard/2004a  Rivard/2004b | a) Staff training and consultation [Rivard/2003, Rivard/2004b]^15,31,41b,43,55,65^  b) Booster trainings[Rivard/2003, Rivard/2004b]^19,56^  c) Unit mission statements[Rivard/2003, Rivard/2004b]^52^  d) Core team [Rivard/2004b]^33,35^  e) Initial pilot [Rivard/2004a, Rivard/2004b]^61^  f) Partnership with academic institution [Rivard/2004b]^24^ | a) Training: Staff “are trained in the basic principles of the Sanctuary Model and taught how to diffuse the model into the environment and into all aspects of  the treatment program”[ Rivard/2004b]^1,3^  Consultation: “To translate the Sanctuary Model philosophy, principles, and language into daily programming, team meetings, treatment planning, community meetings, and work with families”[Rivard/2003, Rivard/2004b]^3^  b) “In response to identified needs [for more training]”[Rivard/2003]^7^  c) To begin “diffusion of this enhanced therapeutic community philosophy”^6,8^ [Rivard/2004b]  d) To guide initial program development and pave the way for implementation^7^  e) NR  f) “To develop evidence-based practices.” [Rivard/2004b]^7^ |
| Sanctuary Model | Rivard/2005 | a) Staff training, staff dialogues and on-going technical assistance^8,15^  b) Self evaluations of residential units’ structure and functioning, Implementation Milestones Checklist^27^ | a) NR  b) NR |
| **Sensory Integration Initiatives** |  |  |  |
| Trauma-Informed Care (TIC) and Ayres Sensory Integration Training | Denision/2018 | a) Inservice training^15,31^ | a) To impact the knowledge and attitudes of staff members^1,2^ |
| Massachusetts State R/S Prevention Initiative: Integrating Sensory and Trauma-Informed Interventions | Lebel /2004  Lebel/2010 | Sensory Initiative:  a) Funding from SAMHSA^1^  b) Partnering with national R/S reduction initiative – sensory based crisis planning tool^30,52^  c)State-wide training^15,16,19,71^  d) Sensory rooms and equipment^11^  e) Crisis prevention toolkit, resource guide, and educational curriculum^29^  f) New state regulations: Trauma-informed, individualized, sensory-based treatment and crisis plans^44,77^  g) Statewide surveys of inpatient and intensive  residential treatment providers^56^  Broader Initiative:  h) Evaluating data as part of a quality management process^27^  i) Review of actions by other State Mental Health Authorities^7,72^; presented at roundtable discussions^17^  j)Departmental mandate to reduce/eliminate restraints and seclusions^44^  k) Training, consultation, and technical assistance^15,54,55,65^  l) Use of the Safety Tool^30,41a,44,51^  m)Roundtable discussions^52^  n) Conferences^7,15,41a,43^ and grand rounds series^7,15,33^  o) Strategic plans^4,23,51,54^  p) Manual under development^29^ | a) “to actualize seclusion-and-restraint reduction initiatives.”^3^ [Lebel/2010]  b) NR  c) Contracted OTs “to provide a statewide training for occupational therapy and allied health professionals to help spearhead a sensory modulation train-the-trainer initiative”^1,8^[Lebel/2010]  d)NR  e) ”To support new learning and practice”^1,3 “^To inform and advance the statewide effort”^7^[Lebel/2010]  f) “To prevent the use of R/S and [to mandate] requirements if they are used”^3,8^ [Lebel/2010]  g) “To support the development of sensory-based practices”^7^ “To assess the extent of sensory intervention interest, implementation, and outcomes”^7^ [Lebel/2010]  h) “To measure and compare its [each unit’s] performance against similar units statewide”^7^ [Lebel/2004]  i) NR  j) NR  k) “To help the provider community meet the R/S [restraints/seclusions] reduction goal”^7^ [Lebel/2004]  l) NR  m) “Offered peer-to-peer support and encouraged collective problem solving to change culture and implement innovative R/S [restraints/seclusions] reduction approaches.”^7,8^ [Lebel/2004]  n) Grand Round Series 1: “helped providers refine their strategic plans and strength-based approaches”^7^ ; Grand Round Series 2: “To link DMH [Department of Mental Health] efforts with those of other child/adolescent-serving state agencies and to enhance supports for children and adolescents with trauma histories”^3,7^ [Lebel/2004]  o) “To introduce strength-based care and reduce R/S [restraints/seclusions]”^7^ [Lebel/2004]  p) NR |
| Sensory Modulation and Trauma-Informed-Care | McEvedy/2017 | a)Train the trainer training^1,15,19,31,43,47,51,71^ | a) “equipping trainees with knowledge and confidence to educate their nursing, medical and allied health colleagues (end-users)”^1^; “to translate newly acquired knowledge into practice by adopting SM and TIC strategies^3^, with a view to these becoming embedded as part of routine care in mental health service delivery.” ^8^ |
| Sensory Room/Occupational Therapy (OT) Consultation/Sensory Motor Arousal Regulation Treatment (SMART) | Warner/2013 | Cohannet Academy:  a) Sensory diet and sensory rooms^11,41b^  b) Tracking target measures^27^  OTA Watertown: Brandon:  c) Funding to hire OTs to train all staff and provide therapy^1,21,57^  d)Structural changes on the unit and residences^11,41a,65^  e) Referrals for full extent of sensory integration based OT^74^  OTA: Gifford School  f) OT consultation model^65^ including an OT room^11^  g) Training by the OT for staff^15^  OTA: GLC  h) Contract with OTA Watertown^34,65^  SMART:  i) SMART rooms^11^  j)Pilot project^61^  k)Training therapists^15,43,65^  l)Videotaping of therapy sessions^26,74^  m)Therapist supervision^53^ | a) “To expand sensory modulation interventions offered in the program”^3^  b) NR  c) “To hire occupational therapists to train all therapeutic, educational and residential staff in sensory integration theory and sensory modulation techniques and strategies”^1,3,7^  d) NR  e) “To address sensory modulation needs, sensory discrimination, postural, ocular and bilateral motor coordination problems, as well as praxis”^7^  f) “To use the OT room as a place to learn what strategies are most beneficial and then to utilize these strategies in the classroom or break rooms.”^3,7^  g) NR  h) “To develop a program to address sensory modulation difficulties”^7^  i) “To allow for movement and utilization of some basic equipment”^7^  j)NR  k) “The goal was to share tools regarding sensory modulation for arousal regulation, a part of sensory integration that might practically work in psychotherapy”^1,3^  l) ”Caregivers or guardians are given a consent form that indicates options for use of videotape for clinical, supervisory, teaching and/or research purposes”^7^  “The SMART team continued to view videotapes together with the OT to identify the kinds of sensory motor input that seemed to be upregulating and down-regulating for individual clients in order to feed back to the clinicians in their consultations.”^7^  m)NR |
| **Structured Psychotherapy**  **for Adolescents Responding to Chronic Stress (SPARCS)** |  |  |  |
| SPARCS | Habib/2013 | a) Training and supervision^15,51,53,65^  b) Consultation^55,65^  c) Sustainability: ongoing in-house trainings, staff meetings^19^, email reminders and updates, posters^31,58^ | a) To “assist clinicians in flexibly applying material in a manner that is both personally relevant to the group and maintains fidelity to the core concepts”^3^  b) “for assistance with recruitment, assessment, engagement, retention, and treatment implementation”^7^  c) “ways for spreading concepts and techniques throughout a system”^8^ |
| **Trauma Affect Regulation: Guide for Education and Therapy (TARGET)** |  |  |  |
| TARGET and environmental modifications | Marrow/2012 | a) General trauma training^15,44,52^  b) TARGET training, supervision, and consultation^15,53,55^  c) Environmental modifications^11,41ab,51^ | a) To increase knowledge on childhood traumatic stress^1^; To plan and design ways to integrate trauma-focused interventions onto the unit^7^  c) To “assist in reducing noise and other triggers and … allow spaces for youth to practice coping skills”^8^ |
| **Trauma-Informed Psychiatric Residential Treatment (TI-PRT)** |  |  |  |
| TI-PRT | Boel-Studt/2017 | a)Hire and designate personnel to lead the process^57^  b)Training and supervision^15,19,41b,53^  c)Training and implementation checklist ^27^  d) Trauma education for families^41a,50^ | a) To convert the agency to a trauma-informed organization^8^  b) To understand trauma and working effectively with trauma-affected youth^1,3^  c) To assess fidelity to the model^7^  d) “To provide trauma education and teach skills to help them support their child’s treatment.”^1,3,5^ |
| **Trauma-Systems Therapy (TST)** |  |  |  |
| TST | Brown/2013  Murphy/2017  Redd/2017 | All three sites:  a) Commitment from leadership ^44^  b) Training staff at all levels^15^  c)Development of tools^26^  The Children’s Village:  d) Tracking clinical outcomes^27^  e) Piloting the model^61^  KVS Health Systems:  f) ) Partnership and creation of fidelity measures^26,65^  g) Trainings, workbooks, newsletters, consultation calls, coaching and supervision.^15,16,19,29,31,41a,43,50,53,55,65,69^  h) Integrating training feedback from interviews and focus groups^46b^  i) Sustainability team^48^  j) Peer coaches^35^  k) Foster/resource parent training coaching, and mentoring and birth parent training^2,15,29,31,33,41a,43,44,50^  l) Planning and implementing evaluation and fidelity feedback^5,24,26,27,51^  m) TST tools and assessments^74^  n) Enhanced centralized data system^12,56^  o) Community partners training^6,76^  p) Piloting the model^61^  Adaptation by one unidentified agency:  q) Integrating training into existing meetings ^48^ | a) “To make changes throughout every level of the organization.”^4^ [Brown/2013]  b) To create “a TST team of which direct care staff are a crucial component”^6^ [Brown/2013]  c) To assess the youth’s trauma system and to assess the functioning of the residential team/milieu^3^  d) To use “this data to improve treatment”^3^[Brown/2013]  e) NR  f) “To develop and provide a wide range of training approaches  (including web-based e-learning modules) specific to unique roles of  individuals that comprise children's care teams”^7^ [Murphy/2017]  g) ”To ensure that youth leaving residential care received the same, consistent, child-specific TST services in the community upon discharge”^1,3^ [Brown/2013]  h) NR  i) To provide “oversight to the TST implementation effort”^7^  j) “To serve as a peer coaches or models for newly hired staff. These peer  coaches provided direct support for staff on how to use TST and related  tools effectively within their roles”^3,7^ [Redd/2017]  k) Multiple modes of training: “To boost participation rates of resource parents in the TST training”^5^ [Redd/2017]  l) To evaluate TST^7^  m) “To provide tools to help staff and foster parents apply their knowledge of TST into daily practice”^3,5^ [Redd/2017]  n) “To better monitor implementation of TST”^7^ [Redd/2017]  o)NR  p) NR  q) To enable direct care staff to participate in trainings^7^ |
| **Uncategorized TIC Programs** |  |  |  |
| Gender-Specific and Trauma-Informed Training Curriculum | Crable/2013 | a)Training program^15,16,65^ | a)“To increase awareness of best practice interventions^1^; improve understanding of components of trauma-informed care^1^; improve engagement skills with youth^3^; improve emotional and physical boundaries with youth^3^; improve understanding of cycle of sexual retraumatization^3^” |
| Trauma-Informed Approach (TIA) | Craig/2018 | a) Leadership and communication components^32,44^  b) Training^15,44^  c) Employee feedback^46b^  d) Measurement^27^  e) Debriefing^33^  f) A written Implementation plan^23^  g) Support component^39b,60^  h) Creating a treatment team^21^  i) Intervention in accordance with Joint  Committee on Standards for Educational Evaluation^77^ | a)"To create a vision that sets a tone for future success”, To provide a clear vision and passion for the initiative, ”identifying different vehicles to communicate the message, modeling practices, and sharing results and progress routinely”^1,3^  b) “teaching the knowledge and skills needed to practically implement a philosophy of comfort-verses-control…”^1,3^ “To share the motivation behind the initiative “^1^  c) “To ascertain how employees felt about the change”^7^  d) "To evaluate whether progress was being made to guide subsequent steps in the improvement process.”^7^  e) “To determine whether each use of restraint or seclusion was warranted or unwarranted”^1^, “To avoid future use of restraint or seclusion”^3^, “to review the antecedents to avoid, and the supports needed to prevent future restraints or  seclusions”^3,7^  f) “To establish accountability as to who was responsible for what, where, and when”^3^  g) To provide support^3^, “allowing for modelling and teaching opportunities”^3^  h) To implement therapeutic treatment planning^3^  i) NR |
| Trauma-Informed Care (TIC) Program | Jacobowitz/2015 | a) Regularly scheduled TIC meetings for all levels of direct care staff^15^ | a) To discuss “the principles of TIC and a specific patient’s or multiple patients’ behaviors”^1^ |
| Trauma-Informed Child Welfare Service (CWS)/ The Connecticut Collaborative on Effective Practices for Trauma (CONCEPT) | Lang/2013 | a) Funding and collaboration^1,6,24^  b) Aligning initiative with government mandate^77^  c) Core team and subcommittees^48^  d) Workforce development: trauma champions^19,35^, mandatory trauma training^15,19,44,71^, addressing worker wellness and traumatic stress^2,7,11,15,21,41b,44,48^  e) Trauma screening^12^  f) Trauma-informed policy and practice guide revisions^44,64,74^  g) EBP dissemination^20,55,74,76^  h) System-level evaluation procedures^27^ | a) “To support development of trauma-informed CWSs”^7^  b) NR  c) To provide “oversight of planning and implementation for CONCEPT”^7^  d) Trauma Champions: “to provide at least one monthly in-service training focused on trauma in their local office”^1^; Trauma Training: “To improve knowledge about child trauma and promote trauma-informed practice change….”^1,3^; Train the trainer: “To promote sustainability”^8^; Officewide staff events: “to promote peer supports and improved morale”^6^  e) NR f) “To align department policy with its practice efforts to create a trauma-informed CWS”^4^; “with the goal of supporting trauma-informed care”^8^  g) To increase “availability of trauma-focused EBPs”^3^  h) “to assess system level change in the overall capacity of the CWS to deliver trauma-informed care”^7^ |
| Trauma-Informed Care (TIC) Training Program | Williams/2017 | a) Funding from the Western Australian (WA)Mental Health Commission^1^  b) Academic partnership^24,27^  c) Training program (separate trainings for clinicians and for managers)^15^ | a) To fund the TIC training program^7^  b) “To inform a business case for further funding to continue the training programme.”^7^  c) “The training was specifically designed to enable the adoption of trauma informed approaches throughout public mental health and alcohol and other drug services in Western Australia.”^3^  Clinician training: “To equip them with basic knowledge and skills which could be adapted and applied to their roles within their services.”^1,3^  Manager training: “to support them in implementing TIC principles within their services.”^7^ |

Table S3.

*ERIC strategies and overarching themes*

| **Classification/Theme** | **Strategy within Theme** |
| --- | --- |
| **A. Use evaluative and iterative strategies** | E4 Assess for readiness and identify barriers and facilitators; E5 Audit and provide feedback; E56 Purposefully reexamine the implementation; E26 Develop and implement tools for quality monitoring; E27 Develop and organize quality monitoring systems; E23 Develop an implementation blueprint; E18 Conduct local need assessment; E61 Stage implementation scale up; E46 Obtain and use a) patients/consumers and family or b) staff feedback; E14 Conduct cyclical small tests of change |
| **B. Provide interactive assistance** | E33 Implementation Facilitation; E54 Provide local technical assistance; E53 Provide clinical supervision ; E8 Centralize technical assistance |
| **C. Adapt and tailor to context** | E63 Tailor strategies; E51 Promote adaptability; E67 Use data experts; E68 Use data warehousing techniques |
| **D. Develop stakeholder interrelationships** | E35 Identify and prepare champions ; E48 Organize implementation teams and team meetings; E57 Recruit, designate, and train for leadership; E38 Inform local opinion leaders; E6 Build a coalition; E47 Obtain formal commitments; E36 Identify early adopters; E17 Conduct local consensus discussions; E7 Capture and share local knowledge; E64 Use advisory boards and workgroups; E65 Use an implementation advisor; E45 Model and simulate change; E72 Visit other sites; E40 Involve executive boards; E25 Develop an implementation glossary; E24 Develop academic partnerships; E52 Promote network weaving |
| **E. Train and educate stakeholders** | E19 Conduct ongoing training; E55 Provide ongoing consultation; E29 Develop educational materials; E43 Make training dynamic; E31 Distribute educational materials; E71 Use train-the-trainer strategies; E15 Conduct educational meetings; E16 Conduct educational outreach visits; E20 Create a learning collaborative; E60 Shadow other experts; E73 Work with educational institutions |
| **F. Support clinicians** | E32 Facilitate relay of clinical data to providers; E58 Remind clinicians; E30 Develop resource sharing agreements; E59 Revise professional roles; E21 Create new clinical teams |
| **G. Engage consumers & staff** | E41 Involve a) patients/consumers and family members or b) staff; E39 Intervene with a) patients/consumers or b) staff to enhance uptake and adherence; E50 Prepare patients/consumers to be active participants; E37 Increase demand; E69 Use mass media |
| **H. Utilize financial strategies** | E34 Fund and contract for the clinical innovation; E1 Access new funding; E49 Place innovation on fee for service lists/formularies; E2 Alter incentive/allowance structures; E42 Make billing easier; E3 Alter patient/consumer fees; E70 Use other payment schemes; E28 Develop disincentives; E66 Use capitated payments |
| **I. Change infrastructure** | E44 Mandate change; E12 Change record systems; E11 Change physical structure and equipment; E22 Create or change credentialing and/or licensure standards; E13 Change service sites; E9 Change accreditation or membership requirements; E62 Start a dissemination organization; E10 Change liability laws |
| **J. Not categorized** | E74 Assess and redesign workflow; E75 Create online learning communities; E76 Engage community resources; E77 Align with organizational or government mandate |

Table S4.

*ERIC strategies and overarching themes used with each TIC intervention.*

*See Table S3, above, for the legend corresponding to the ERIC themes and categories.*
