# Supplementary material for: Trauma-Informed Care Interventions Used in Pediatric Inpatient or Residential Treatment Mental Health Settings and Strategies to Implement Them: A Scoping Review
Source: Trauma Violence Abuse. 2023 Sep 11;25(3):1737–55. doi: 10.1177/15248380231193444 (PMC11155220; doi:10.1177/15248380231193444)
Supplement: sj-docx-3-tva-10.1177_15248380231193444 – Supplemental material for Trauma-Informed Care Interventions Used in Pediatric Inpatient or Residential Treatment Mental Health Settings and Strategies to Implement Them: A Scoping Review [file sj-docx-3-tva-10.1177_15248380231193444.docx]

**Supplementary File A**

Table S1.

*TIC Interventions Grouped by Intervention (n=21)*

| **Intervention Name** | | **Author/ Year** | **Aims of Intervention (A)** | **Ingredients (I)** | **Mechanism (M) or Theoretical Basis** | **TIC Delivery (D)** | | |
| --- | --- | --- | --- | --- | --- | --- | --- | --- |
| **Attachment, Regulation and Competency (ARC)** | |  |  |  |  |  | | |
| Project Penguin informed by ARC and Positive Behavioural Interventions and Supports (PBIS) | | Brend/2020 | “To respond to the needs of residential treatment centres serving children aged 6 to 12”^C^  “To help workers develop trauma-informed attitudes and implement trauma-informed practices^B^, make the workplace more responsive to the well-being of RC [residential childcare] workers^D^, and reduce the use of restraints and seclusions among school-aged children in RCs^A^” | - Tier 1: Competency, emotion regulation, routines, and rhythms: These elements of Program Penguin are in place for all children at all times - Tier 2: Strategies employed for children who are at a higher risk or of higher need - Tier 3: Individual interventions and referrals: For the most vulnerable children or those who are not responding to the interventions represented in the first two tiers. - Adapted adolescent program (Program Polaris) | Four distinct yet overlapping knowledge bring Program Penguin together.   - Understanding Complex Trauma - Attachment Self-Regulation and Competency (ARC) - Positive Behavioural Interventions & Support (PBIS) - Social Innovation (Implementation) | - Tier 1 example: “several units created calming rooms” | | |
| Building Communities of Care (BCC) | | Forrest/ 2018 | “To create a system-wide community of care that maximizes client growth and capacity for self-regulation^E^ while minimizing violent incidents^A^ and costs for the organization.”  “BCC was designed to reduce the frequency and intrusiveness of restraints.”^A^  “To create a restorative community”^F^  “To collaboratively design, implement, and maintain an environmental culture^F^ where instances of client dysregulation  and difficulty become a rare occurrence” | - Integrative: Intentionally coordinated programming across systems with a common language - Individualized: “Caregivers [providers] maintain routines, policies, and systems upon which they individualize care with a menu-based approach, including collaboratively developing treatment plans, individualizing the environment for maximum predictability, comfort, and safety, and planning youth-selected activities.” - Proactive: “Caregivers [providers] are trained to attune to initial signs of dysregulation and respond with validating strategies to deescalate before a negative strategy [restraint] is needed.”   “BCC is broken down into core considerations of trauma-informed care: the environment, clinical treatment, community engagement, and behavioral interventions. These core considerations exist across three ecological systems: individual, community, and external” | - “BCC is grounded in the empirically supported Attachment, Regulation, and Competency (ARC) Model” - “The ARC foundation constitutes enhancing children’s caregiver-child relationships (attachment), skills to manage internal, and interpersonal experiences (regulation), and key capacities associated with resilience (competency).” | - Individualized: “For example, bedrooms are decorated prior to clients’ arrival with the interests of each client in mind, while simultaneously eliminating possible triggers of dysregulation. Additionally, during intake parents and their children approve the types of non-verbal, verbal, and physical interventions appropriate if necessary” - Proactive: “Attuning includes remaining aware of how caregiver body language and facial expression affects the situation, and maintaining a nonthreatening approach. In the event a restraint occurs, caregivers [providers] utilize the least intrusive, preapproved restraint while remaining attuned to the child’s needs and level of dysregulation so release occurs promptly. Post-restraint a debriefing period involves finding the dysregulation and escalation triggers in order to remove them as well as addressing potential retraumatization.” | | |
| ARC; Grow Strong/Stepping Stones | | Hodgdon/ 2013 | To “offer a guiding structure for providers working with trauma-impacted youth and their care- givers,^B^ while allowing significant flexibility in application” | ARC is a framework for intervention with youth and families with Three Core Domains: Attachment, Self-Regulation, and Competency   - “Within these domains, nine core targets or “building blocks” of intervention are delineated, along with a tenth target, Trauma Experience Integration” - Milieu/program culture changes - IRTP site:   - ARC individual therapy   - ARC group therapy: “Grow Strong” - Residential school site   - “Stepping Stones Program”: Individual and group therapy   - Incorporating techniques, psychoeducation, and activities from several treatment models including ARC, Structured Psychotherapy for Adolescents Responding to Chronic Stress (SPARCS), and Urban Improv-Intensive Protocol for Middle School Children Exposed to Trauma and Violence.” | - Changing “the intervention strategy from punishing unwanted behavior to teaching and supporting alternative skills in order to increase a client’s sense of control and mastery.” - “Stepping Stones”: “The overall principles of the individual component include: being developmentally tailored, self-enhancement focused, integrates consistent response and routines and rituals, and co-regulation of affective states.” | ARC Individual:   - “At IRTP, specific clients were selected by their clinicians to participate in ARC individual treatment... clinicians implemented an explicit ritual with regulation strategies practiced at the beginning and end of session…all ARC individual clients received psychoeducation about triggers and the trauma response. Specific treatment targets within the framework were selected based on individualized assessment of client needs…In addition, a group of clients … were selected for an ARC group. Eligible clients were asked to voluntarily participate and therefore self-selected into the treatment. The ARC group, named “Grow Strong” was a 16 session group…The group was co-facilitated by the trauma consultant and clinical director.” - “Each ARC group followed a structure with the following components presented in a specific order: 1) self-regulation exercise, 2) self-appraisal and rating of physiological/ emotional response to the exercise, 3) snack, 4) homework review, 5) psychoeducation about a specific ARC skill, and 6) self-regulation exercise followed by self-appraisal and rating of emotional response...homework was an integral part of each group to support ongoing practice and generalization of skills to the milieu and community settings.” - “Self-regulation exercises were incorporated into all sessions of the group”   Stepping Stones Program:   - “With both individual and group programming… incorporating techniques, psychoeducation, and activities from several treatment models including ARC… - “clients were deemed eligible for the group based on a history of successful participation in group treatment and were asked to voluntarily participate….”   Stepping Stones Group:   - “A 22 session group … Each group session had specific components including: 1) initial check in and self-appraisal rating of distress and control, 2) mindfulness activity, 3) snack, 4) review of prior week, 5) psychoeducation/content, 6) experiential activity, 7) sharing of progress with personal goal, 8) homework review, and 9) ending self-appraisal using same distress/control scale at used at the start of group.” - “Stepping Stones addressed self-regulation, cooperative relational engagement, and competency building skills in each session, offering repetitive experiential practice of skills, techniques, and activities...Homework was also an integral part of each group…as well as the integration of several community building activities that were implemented in milieu setting.”   Stepping Stones individual intervention:   - “Clients who participated in the Stepping Stones group also received the Stepping Stones individual intervention.” - “Each session had a specific structure, starting with a feelings check-in, mindfulness activity, goal activity from the session (client could choose from a group of activities, all of which would be covered throughout the course of the treatment).”   Milieu/program culture:   - “Teaching and supporting alternative skills” - “Clearly defined structure that includes programmatic rules and expectations and associated rewards/ consequences and daily routines. The structure must be explicitly stated and accessible through visual cues posted within the milieu” - “ARC-based milieu activities were designed to parallel and support clinical group and individual intervention on multiple fronts, including initiatives such as creating psychoeducational bulletin boards in the residential common areas and classrooms, changing behavior systems” - “One program created “the comfort zone” on the unit… to be used by clients (with the support of staff) to practice various up- and down-regulation skills”   “Grow Strong” group:   - “The group was co-facilitated by the trauma consultant and clinical director.”   Stepping Stones individual intervention:   - “Following every group session, each client would meet with his or her individual therapist for a 1-h session focused on the components of the group ending with a grounding exercise and review of the session.” - “One program created an “On Track Action” wall… The goal of the wall was to provide clients with weekly and monthly incentives for positive behaviors and to provide staff members with a systematic tool for consistently giving praise” | | |
| Trauma-Informed Care (TIC) Training informed by the Substance Abuse and Mental Health Services Administration (SAMHSA) and ARC | | Matte-Landry/ 2021 | NR | - Incorporating TIC in day-to-day interventions and procedures - Specific actions that could be used by staff to de-escalate crises and to minimize the use of restrictive measures - Principles and intervention targets from SAMHSA, ARC and PBIS | - Training material informed by the principles introduced by SAMHSA and the ARC framework - “An adapted version of this training was created for workers serving children in 6 of the 15 community group homes in order to integrate Positive Behavioral Interventions and Supports (PBIS)” | Not described** | | |
| **Child Adult Relationship Enhancement (CARE)** | |  |  |  |  |  | | |
| Child Adult Relationship Enhancement (CARE) | | Gurwitch/ 2016 | To fill the void of “services^C^ to enhance the child–adult relationships for these children, and potentially reduce risk for maltreatment or other behavioral and relationship concerns”  ”To fill a void often overlooked by large training initiatives – individuals in non-clinical positions who interact with children in health settings, yet often lack the resources and training to implement theoretically-sound and practical behavior management skills.”^B^  To be a “prevention model for children at risk for maltreatment or other behavioral concerns, or to complement ongoing therapy services.”^C^ | - CARE training (contains a trauma education component)* - CARE skills   - The “P”s: Praise, Paraphrase, Point out   - The “Q”s: Quash, Quit, Quiet   - Strategic ignoring - Good commands and directions | - Parent Child Interaction Therapy (PCIT) forms the basis of CARE - “If adults learn ways to quickly and effectively engage in a positive relationship with the child or adolescent, CARE developers theorized that the children and adolescents will be more likely to interact with the adult in an appropriate manner, including improving compliance.” | - CARE skills can be implemented by any adult interacting with a child between the ages of 2 and 18 years of age. | | |
| **Children and Residential Experiences (CARE)** | |  |  |  |  |  | | |
| Children and Residential Experiences (CARE) | | Izzo/2016 | “A principle based program that helps agencies use a set of evidence informed principles to guide programming^C^ and enrich the relational dynamics throughout the agency^F^.”  “Designed to enhance the social dynamics in group care settings through targeted staff development and ongoing reflective practice” | - Six evidence informed principles:   - Relationship-based   - Trauma-informed   - Developmentally focused   - Family-involved   - Competence-centered   - Ecologically oriented | - “CARE explicitly uses an ecological approach to help agencies transition from simply maintaining compliance to creating a living environment that offers/provides developmentally enriching experiences and a ‘sense of normality’ “ | NR | | |
| **Collaborative Problem Solving (CPS)** | |  |  |  |  |  | | |
| CPS | | Greene/ 2006  Martin/ 2008 | 1- “to help adults identify the cognitive factors that may contribute to aggressive outbursts of children and adolescents, most notably in the domains of emotion regulation, frustration tolerance, problem solving, and adaptability skills.”^C^  2- “to help adults become cognizant of three common options for handling problems or unmet expectations— imposition of adult will, collaborative problem solving, and removal of the expectation—and the impact of each of these three strategies on adult-child interactions.”^B^  3- “to help adults^D^ and children^E^ become proficient at solving problems collaboratively so as to resolve potentially conflictual situations in a manner that reduces the likelihood of aggressive outbursts, facilitates assessment of the cognitive factors underlying the child’s difficulties^C^ (in our view, this is among the foremost goals of an inpatient stay), and length of stay permitting, teaches the child specific cognitive skills^E^.” [Greene/2006]  “To comprehensively assess—and ultimately teach—specific cognitive skills^C^ that may be contributing to difficulties in these global domains.” [Greene/2006]  To work with “children and adolescents with oppositional behaviours and aggressive outbursts” [Martin/2008]  “To identify pertinent social and cognitive pathway impairments and precipitating antecedent events^C^… Through these means, CPS seeks to ultimately prevent further aggressive outbursts.” [Martin/2008] | - Assessment of lagging skills   - Executive functioning (including inattention, disorganized thinking, and poor handling of transitions),   - Language processing (such as expressive or receptive impairments and difficulty expressing feelings),   - Emotion regulation (including irritability, anxiety, and distorted self-perception),   - Cognitive flexibility (such as concrete thinking and insistence on sameness and rigid routines), -social skills (such as misreading interpersonal nuance and difficulty appreciating the views of others). - Skills training | - Rooted in cognitive behavioural principles - “Implementation of the model also typically leads to contemplation of unit organization, schedule, structure, and expectations that may actually heighten the likelihood of aggressive outbursts. Adoption of the model results in improvement in mechanisms for staff communication and significant augmentation of the role of direct care staff, from mere “behavior managers” to active participants in each resident’s assessment and treatment.” [Greene/2006] | - “Assessing and teaching skills primarily in the context of ongoing staff-resident interactions rather than in cognitive skills groups.” [Green/2006] | | |
| CPS | | Pollastri/ 2015 | To understand and intervene with youth who have social, emotional, and behavioural challenges. ^C^ | - Understanding unmet expectations as lagging skills - Assessing lagging skills - Intervening with 3 plans: Plan A, Plan B, Plan C | - “The guiding philosophy of CPS posits that “kids do well if they can,” and youth who are often thought of as disruptive, oppositional, and challenging are re-conceptualized as lacking the skill, not the will, to behave well.” - CPS is thought to increase caregivers’ empathy and patience, resulting in reduced use of restrictive practices and improved youth-adult relationships - CPS is thought to improve cognitive skills in youth resulting in improved behavioural and emotional symptoms in youth | - “The collaborative problem solving process can take place between a CPS-trained adult and a youth whenever the adult recognizes that the youth is having consistent difficulty meeting expectations, responding to triggers, or following rules in a home, school, or community setting. Any adult caregiver can become trained in Collaborative Problem Solving; understanding and implementing the model does not require advanced clinical training.” | | |
| Child and Family Centered Care (CFCC): CPS, Open Hours, and Trauma-Sensitive Protocols | | Regan/2010  Regan/2017 | To move “from a traditional consequence based milieu to one that was collaborative, nurturing, and child and family centered”^F^ (Regan, 2010)  To decrease restraints and seclusions^A^ and to increase a sense of safety and staff work satisfaction^D^ (Regan/2017)  CPS: “to understand and help children manage the frustration and the cognitive underpinnings that generate behavioral dyscontrol”^C^ (Regan, 2010)  CPS: “To provide a child-centered model of care around child behavioral problems” (Regan, 2017)  Open hours: “to join with parents in creating partnerships to care for the children admitted to the unit”^C^ (Regan, 2017) | - Adoption of collaborative problem solving (CPS) model in responding to children's behavioral difficulties - Open hours for parents - Trauma-sensitive protocols and procedures. - Nursing Code of Ethics with interpretive statements* | - “CPS is based on the assumption that children do well if they can.” (Regan/2017) - Principals:   - dignity and respect   - affirming and useful communication   - strengths- based and participatory   - collaboration | - CPS: “Under the CPS approach, a staff member approaches the child with a desire to understand the child's refusal to attend group and to learn all he/she could from the child… The staff person's agenda is to prevent a “meltdown” (an aggressive behavioral escalation), to maintain the child's trust by showing concern (expressed through empathy), and to resolve the problem in a mutually satisfactory manner, taking into account the concerns of both child and adult. Again, the child is not forced to attend the group, nor is he/she given a consequence for not going.”(Regan, 2017). - Open hours policy:” Parents may come to the unit at any time, for any length of time.” - Trauma-Sensitive: “Positive physical touch from safe, stable adults is considered an important component in helping children heal and in providing a “safe haven.””; “(1) children may sleep in the hall in view of staff if they wish; (2) they may stay up until tired if they are afraid to enter the bedroom; (3) some children who are fearful of their own thoughts at bedtime and become agitated are permitted to watch TV in their room until sleepy.” - “Medical procedures and protocols are not forced on children” | | |
| **Devereux’s Safe and Positive Approaches (SPA)** | |  |  |  |  |  | | |
| SPA | | Russell/ 2009 | “To equip staff with the knowledge and ability to safely and effectively prevent, de-escalate, and manage crisis situations.”^B^ | The SPA program has three components:   - Staff Effectiveness Training* - Safety Techniques Training* - Personal Emergency Interventions Training* | NR | NR | | |
| **EQ2: Empowering Direct Care Staff to Build Trauma-Responsive Communities for Youth** | |  |  |  |  |  | | |
| EQ2 | | Griffing/ 2020 | To address “staffs’ understanding of the effects of complex trauma on youths’ social, emotional and cognitive development and functioning”^B^  To build “staff’s self-regulation and social-emotional skills to promote resilience, reduce burnout^D^ and create a more trauma- responsive community^F^.”  “To create an ongoing support for staff to serve as a buffer against secondary traumatic stress.”^D^  To provide “skills to manage conflict that can arise in the milieu^B^, to create a psychologically safer environment for staff and clients^F^.” | - Trauma-informed knowledge - Mindfulness-based practices (e.g., attention training, focused-breathing exercises, and guided visualizations reinforcing session content)* - Practices from restorative justice (e.g., Circles)* - “skills to manage conflict that can arise in the milieu” | - “These skills are essential if staff are to create environments in which they can form and maintain nurturing and reparative relationships with youth, because self-regulation skills necessarily precede co-regulation skills” - “EQ2 encompasses several key components of a trauma-informed approach as outlined by Substance Abuse and Mental Health Services Administration (SAMHSA, 2014), most notably: 1) building safety; 2) increasing trustworthiness and transparency amongst staff; 3) promoting peer support; 4) enhancing collaboration and mutuality; and, 5) bolstering staff empowerment, voice and choice.” | Not described | | |
| **NASMHPD Six Core Strategies** | |  |  |  |  |  | | |
| NASMHPD Six Core Strategies | | Azeem/ 2011 | To reduce “the use of seclusion and restraints with hospitalized youth”^A^ | - Leadership towards organizational change* - Use of data to inform practice* - Workforce development* - Use of restraint and seclusion reduction tools - Improving consumers’ role in inpatient setting* - Vigorous debriefing techniques.* | - Trauma-informed and strength-based care “with a focus on primary prevention principles “ | - Use of Restraint and Reduction Prevention tools: “Using primary prevention principles, a variety of tools and assessments were included in the individual treatment plans.” | | |
| NASMHPD Six Core Strategies (and Building Bridges Initiative; BBI) | | Azeem/ 2015  Caldwell/ 2014 | “To reduce the use of restraints^A^ and to provide trauma informed care”^C^[Azeem/2015]  To create a culture change of the milieus being trauma informed and strength based^F^ [Azeem/2015]  “To prevent the conflicts that lead to violence and the use of R/S [restraints and seclusions] in residential and inpatient mental health programs”^A^ [Caldwell//2014] | - Leadership toward organizational change* - Youth and family inclusion* - Workforce development* - Prevention tools - Debriefing* - Using data to inform practice* | - “The strategic plan toward those goals was built around the framework of trauma-informed and strength-based care which outlined various activities and initiatives which became embedded through leadership of line staff and milieu managers, unit clinical, and leadership teams.” [Azeem/2015] | - Prevention Tools   Solnit [Azeem/2015]:   - - “Families were welcomed to the hospital before arrival of a youth to tour the facility, meet staff, and familiarize themselves with the principles of care and programming…   - The hospital clinical teams visited complex youth transferring from other facilities to begin the process of engagement and ease their transition…   - An individualized treatment plan was developed by the youth’s team using primary prevention principles…[incorporating] youth’s specific interests, hobbies, coping skills, and motivators …Regular revisions to plans were made …   - The occupational therapy consultant was utilized liberally to assess the sensory needs of youth…   - Youths had access to comfort rooms.... Rehabilitation services have been utilized to provide recreational activities such as music therapy, art, pottery, cooking, and swimming ... Various sports were offered…   - Staff…worked with family members to identify strategies …for de-escalation of youth.”   YDI [Caldwell/2014]:   - - “YDI converted some R/S rooms into “comfort rooms.” Shortly after admission, in addition to an MP3 player with music of their choice from YDI’s approved music library, youth began to and still receive an individualized “comfort box” of sensory items.”   - New admissions are told from the beginning, by both staff and youth, “We do not restrain here.”   - Youth who are admitted with a history of restraints are put on a “hug program,” whereby they receive, as much as they can tolerate, “YDI side-hugs” from…the supervisory and administrative staff…   - Extremely dysregulated youth receive scheduled and individualized sensory regulation breaks during the day.   - Youth who have assaulted or been aggressive with a staff are brought into the Intervention Team (a component of the YDI debriefing process for serious incidents), comprised of one or both of the executive directors, the clinical director, the therapist, case manager, and direct care staff, including the staff member who may have been the recipient of the aggression...to resolve the conflict that has occurred and ensure planning to repair the relationship“ | | |
| ‘Broad TIC program’ based on Six Core Strategies and Risking Connections | | Barnett/ 2018 | “To create a broader cultural shift in the agency^F^ so that the staff would understand and respond sensitively^B^ to traumatized youth.” | Not described | - Content/strategies derived from 6 Core Strategies and Risking Connection models | Not described** | | |
| TIC Program based on the NASMHPD Six Core Strategies | | Hale/2020 | “To reduce the use of crisis interventions”^A^  To help staff learn new ways of being present with the patient, and creating a caring and compassionate response, and not just moving to seclusion and restraint.^B^ | - Commitment of leadership team to organizational change* - Using data to inform the practice change* - Developing the workforce/staff* - Using seclusion and restraint reduction tools - Ensuring that patients/family members have input - Using a three-step debriefing process when crisis interventions are used | - “Having a TIC program provided alternative interventions but, more important, brought a heightened level of staff self-awareness when interacting with an aggressive child.... Staff began to use deescalation techniques learned during education and following debriefings and were able to see that this approach was successful in maintaining the dignity and care of the child, while keeping staff safe.... A consensus of understanding developed with the staff that using crisis interventions increased a patient’s risk for retraumatization; and began approaching each patient situation as if the patient, indeed, had a history of trauma, whether or not they did. This became an under- lying assumption of the TIC culture.” | Deescalation techniques that were a part of the seclusion and restraint reduction tools   - Communicating caring with empathy in a calm manner; - Repeating simple messages as needed - Intentional use of non-threatening body language - Approaching the patient one-to-one (as opposed to multiple people hovering) - Listening, and responding to the expressed needs of the patient - Setting clear limits that were simple - Therapeutic use of self: monitoring one’s own body language, speaking in a low and calm voice, using eye contact, expressing a comportment of empathy.   Three-step debriefing process:   - The first included employees involved … to get data before the end of the shift - The second took place between the patient and a staff who was identified as having the best rapport with the patient within 24 hours; The goal was to record the patient’s perspectives - The final debriefing, occurred within 48 hours, included a review of video documentation of the incident, before, during and after. This was led by the Restraint/Seclusion prevention team, included the employees involved and the assigned social worker Psychological safety was assured; private support meetings would occur as needed. | | |
| **Neurosequential Model of Therapeutics**  **(NMT)** | |  |  |  |  |  | | |
| NMT | | Hambrick/ 2018 | To approach clinical work and problem-solving in a developmentally sensitive manner^F^ | - The three major components of this approach:   - Capacity building and mastery of core concepts*   - An assessment process to determine (a) the timing and nature of developmental adversities and resilience-related factors, (b) current functioning in multiple domains (e.g., sensory integration, self-regulation, relational, cognitive), and (c) current relational milieu (i.e., connection to family, community, culture)   - The selection and sequencing of specific educational, therapeutic and enrichment interventions. | - Based on elements of “attachment, the impact of maltreatment and trauma, and emerging concepts in developmental psychology, neuroscience and traumatology” | Not described** | | |
| **Patient-Focused Intervention (PFI) Model** | |  |  |  |  |  | | |
| PFI | | Barnum Goetz/2012 | To build a culture of safety^F^  To reduce/eliminate use of restraints^A^  To reduce staff injuries^A^ | “PFI model embraced trauma-informed care as the core intervention.”  Components:   - Trauma-informed care principles - Aggression management*, - Code event review*, - Leadership involvement*, - Quality feedback*, - Recovery orientation, - Patient assessment, - Education* - Collaboration* | - Patient-centred, strengths-based | Not described** | | |
| **Risking Connection (RC) and Restorative Approach (RA)** | |  |  |  |  |  | | |
| Risking Connection (RC) and Restorative Approach (RA) | | Baker/2018 | To provide “a pathway towards TIC culture change” ^F^ | - RC and RA are curriculum-based trauma training programs, with a focus on helper well-being and managing vicarious traumatization. - RC:   - Leadership consultation*   - Foundational trauma trainings*   - Guidance about embedding TIC in the system*   - Shared language - RA:   - Emphasizes clients doing learning tasks and restorative tasks (e.g., making things right with the harmed party) rather than receiving punitive consequences. | - RA is based on restorative justice principles | Not described** | | |
| Risking Connection (RC) and Restorative Approach (RA) | | Brown/2012 | “A pathway toward TIC culture change in human service organizations including residential treatment”^F^ | - RC training: *   - “Teaches … that childhood trauma experiences derail the trajectory of development in three critical areas—attachment, brain and nervous system… symptoms are adaptations…Since trauma happens in the context of interpersonal relationships, therapeutic relationships are the primary agent of change and healing”   - focus “on vicarious traumatization and counter- transference”: - Agencies C & D:   - Training in the Restorative Approach, a trauma-informed alternative to point and level systems* | - “Based on constructivist self-development theory (CSDT)…[which draws from] attachment theory, relational psychoanalytic theory, developmental psychopathology, theory of cognitive schemas, and social learning theory”. | Not described** | | |
| **Sanctuary Model** | |  |  |  |  |  | | |
| Sanctuary Model | | Bloom/2003a  Bloom/2003b  Farragher/ 2005  McCorkle/ 2005 | “To help systems move out of the rigid equilibrium …thus enabling them to develop the flexibility in leadership, decision making and therapeutic response ….” ^F^ [Bloom/2003b]  To create a trauma-sensitive culture and build the protective factors necessary for the staff and the agency as a whole ^F^ [Farragher/2005]  “For creating or changing an organizational culture in order to more effectively provide a cohesive, more democratic context within which healing from psychological and social traumatic experience can be addressed” ^F^ [McCorkle/2005] | - A shared base of common assumptions created in a “culture of participation and citizenship” - SELF model: Safety, Emotions, Loss, Future   - “Treatment plans, planning conferences, lifespace interviews and therapy sessions all construct dialogue using the language of SELF.” [Farragher/2005] - Community meetings - 5 components [Farragher/2005]*:   - Integration   - Understanding trauma   - Avoiding reenactment   - Fighting rigidity   - Embracing non-violence.   - Staff knowledge of residents’ history[McCorkle/ 2005]   - Safety plans [McCorkle/2005] | The Model is informed by four basic pillars of knowledge:   - the psychobiology of trauma; - the active creation of nonviolent environments; - principles of social learning; and an understanding of the ways in which complex adaptive systems grow, change, and alter their course. - “A trauma sensitive culture is one in which all members feel physically, psychologically, socially, and morally safe; where members of the community manage their emotions appropriately; acknowledge and deal with loss and grief; and focus on creating a positive future. In the Sanctuary Model, this concept is expressed through the acronym SELF” [Farragher/2005] - “Key values include: a communal atmosphere, group meetings, a belief in the therapeutic role of everyone, participatory democracy, shared authority and responsibility” [McCorkle/ 2005] | - Community meetings: Twice daily with staff and residents; “if there is an ‘incident’ on one of our units it is our practice to call the whole community together to address what we could have done and will do in the future to prevent collective disturbances of disrespect, physical aggression, personal property respect, and sexual boundary issues” [McCorkle/2005] - Safety plans: “The child receives assistance writing this plan with her social worker and primary milieu counsellor, and is encouraged to call upon all safe adults in the Sanctuary community for support.” [McCorkle/2005] | | |
| Sanctuary Model | | Clarke/2012  Leigh-Smith/2014 | “To teach the individual within a group context the necessary skills for creating and sustaining non-violent lives”^E^ [Clarke/2012]  To “support healing from trauma, and bring about organisational change”^F^ [Leigh-Smith/2014]  “To create a therapeutic milieu that promotes safety for the entire community, including  all levels of the organisation” ^F^ [Leigh-Smith/2014]  “The Sanctuary Model provides tools to help carers and staff manage their emotions”^D^ [Leigh-Smith/2014]  “To assist in mitigating the impact of stressors associated with trauma, as well as a means for creating safety within this environment” ^F^ [Leigh-Smith/2014] | - Theoretic Foundation - Sanctuary Norms (7 Commitments, SELF) - Sanctuary Tools (Self-care plans, care planning, red flag meetings, community meetings, safety plans, psychoeducational groupwork, self-care plans, team meetings*) - “Pathways have also included Dr Dan Hughes’ PACE (Playfulness, Acceptance, Curiosity and Empathy) [model]”[Leigh-Smith/2014] | - Trauma Theory - Social Learning Theory - Non violent practice - Complexity Theory - “Concepts such as traumatic re-enactment, parallel process, collective disturbance and vicarious trauma, facilitate an understanding of how trauma can manifest in all areas of the out-of-home care community” [Leigh-Smith/2014] | - Community Meeting: The community meetings are used daily…involve asking three questions of each other:   - How are your feeling?   - What is your goal for today?   - Who are you going to ask for help?   - “Pathways staff have implemented the community meeting with their foster carers and families…Staff have also used community meetings on commencement of departmental meetings” [Leigh-Smith/2014] - Safety Plans: “not only used for children and young people but also for the staff and foster carers” [Clarke/2012]; “The plan is worn at all times and is also known by others around the individual, therefore allowing others to assist with de-escalation by prompting the individual to use the strategies within the plan.” [Clarke/2012]; “Pathways staff are expected and encouraged to wear their safety plans at all times. Safety plans are a set of five instructions to help de-escalate the user and maintain safety.” [Leigh-Smith/2014] - Self-care plans: “All staff are encouraged to have a self-care plan…Staff also help their carers to create self-care plans” [Leigh-Smith/2014] | | |
| Sanctuary Model | Esaki/2014 | “To more effectively provide a cohesive context within which healing from physical, psychological, and social traumatic experience can be addressed” ^F^  “As an organizational culture intervention, the Sanctuary Model is designed to facilitate the development  of structures, processes, and behaviors on the part of staff, clients, and the community as a whole that can counteract the biological, affective, cognitive, social, and existential wounds suffered by the victims of traumatic experience and extended exposure to adversity” ^F^ | - Trauma Theory - 7 Sanctuary Commitments (nonviolence, emotional intelligence, democracy, open communication, social responsibility, commitment to social learning, and growth and change) - SELF acronym (safety, emotion management, loss, and future, is used to formulate plans for client services or treatment as well as for interpersonal and organizational problem solving. - Sanctuary Toolkit (10 practical applications of trauma theory, the Seven Commitments, and S.E.L.F) | Not described | | Not described** |  |  |
| Sanctuary Model | | Rivard/2003  Rivard/2004a  Rivard/2004b | “To teach youths effective adaptation and coping skills to replace nonadaptive cognitive, social, and behavioral strategies that may have emerged earlier as means of coping with traumatic life experiences” ^E^ [Rivard/2003]  To “address maladaptive behaviors and functioning that may have developed in response to repeated traumatic experiences“^C^ [Rivard/2003]  “The intervention is aimed at both strengthening the therapeutic community environment^F^ and at empowering youths to influence their own lives and communities in positive ways^E^” [Rivard/2004b]  “To address the special treatment needs of youth with emotional and behavioral disturbances and histories of maltreatment or exposure to domestic and community violence”^C^ [Rivard/2004a] | - The SAGE Recovery Framework (aka SELF): Safety (including physical, psychological, social and moral), Affect modulation, Grieving, Empowerment - Therapeutic community & Community Meetings - Psychoeducation Program [group and milieu] | - “The intervention … is based in social psychiatry, trauma theories, therapeutic community philosophy, and cognitive-behavioral approaches… incorporates knowledge of the developmental needs of youth.”[Rivard /2003] - “The Sanctuary Model rests upon the basic premise that the therapeutic environment is a critical determinant in facilitating the recovery process.” [Rivard/2003] - “A fundamental premise of the intervention is that the treatment environment is a core modality for modeling healthy relationships among interdependent community members.” [Rivard/2004b] | - “The SAGE recovery framework is integrated into the primary therapeutic modalities of the Sanctuary Model, which include the therapeutic community itself, community meetings, and psychoeducation exercises and groups.” [Rivard/2003] - Community meetings occur twice daily; “A protocol is followed in which all community members share feelings, state their goals for the day, ask for specific help from other members in achieving their goals, share successes at the end of the day, and discuss ways to solve community problems.” [Rivard/2003] - 12 session Psychoeducation Group curriculum: “New skills learned in the psychoeducation groups are practiced and reinforced in everyday activities on the unit, and to prepare for home and community passes.” [Rivard/2003]   Group supplemental activities can be used in unit daily programming and staff interactions with you [Rivard/2004a, Rivard/2004b] | | |
| Sanctuary Model | | Rivard/2005 | “The intervention is aimed both at strengthening the therapeutic community environment^F^ and at empowering youths to influence their own lives and communities in positive ways^E^”  “To specifically address the needs of children and youth that have experienced and often reexperienced  the trauma of maltreatment and community violence^C^” | - Milieu adaptations - Trauma Recovery Framework (SELF: Safety, Emotional management, Loss, Future) - Psychoeducation curriculum for youth | - “The Sanctuary Model integrates an enhanced therapeutic community philosophy (Bloom, 1997), trauma theories (Bloom, 1997), and Friedrich’s (1996) recommended child treatment strategies that address post-traumatic symptoms, developmental disruptions, and unhealthy accommodations to traumatic experiences.” - “A fundamental premise of the Sanctuary Model is that the treatment environment is a core modality for modeling healthy relationships among interdependent community members.” | - “The Sanctuary Model is operationalized through… twice-daily community meetings, a range of pyschoeducation exercises that staff use in their daily interactions with youth, and weekly psychoeducation groups to teach knowledge and skills needed” | | |
| **Sensory Integration Initiatives** | |  |  |  |  |  | | |
| Trauma-Informed care (TIC) and Ayres Sensory Integration Training | | Denision/ 2018 | NR | - In service training* | - “The program was based on best practice, the principles described by Malcolm Knowles’ adult learning theory. the TIC model, and Ayres Sensory Integration theory.” | Not described** | | |
| Massachusetts State R/S Prevention Initiative: Integrating Sensory and Trauma-Informed Interventions | | Lebel /2004  Lebel/2010 | “To reduce the use of restraint and seclusion with children and adolescents in psychiatric inpatient units”^A^ [Lebel/2004]  “To foster feelings of safety and support development and engagement in meaningful life roles, routines, and activities”^E^[Lebel/2010]  “To provide a more nurturing, healing, and trauma-informed culture of care”^F^[Lebel/2010] | Key components include [Lebel/2010]:   - Assessing, exploring sensory tendencies and preferences - Creating sensory diets (individual and programmatic) - Using sensorimotor activities and modalities - Modifying the physical environment - Educating caregivers - Use of the safety tool [Lebel/2004] | “This approach addressed:   - “Primary prevention: establishing collaborative, trauma-sensitive, child-friendly, strength-based models of care that rest on focal problem-solving, stress skill development, and de-emphasize deficits and pathology - Secondary prevention: using early intervention techniques that underscore proactive de-escalation and least restrictive developmentally responsive alternatives tailored to the individual - Tertiary prevention: preventing or reversing negative consequences through the use of anticipatory planning… supporting the earliest possible release from restraint, debriefing of staff, and using patient comment forms, all of which minimize the possibility of harm and encourage prevention” [Lebel/2004] - “Key to the state’s work was the infusion of sensory approaches informed by occupational therapy and the allied health disciplines of nursing, recreational therapy, art therapy, and music therapy” [Lebel/2010] | - Safety tool: “Using the tool, a patient, family member (as appropriate), and staff person collaboratively develop a plan, on admission, that identifies preferred strategies for de-escalation and avoidance of R/S, and restraint preferences to consider if restraint becomes necessary.. Programs have adapted the Safety Tool to meet the developmental - needs of children and adolescents.” [Lebel/2004] - Sensory integration: Not described.** | | |
| Sensory Modulation and Trauma-Informed-Care | | McEvedy/ 2017 | “To transfer knowledge of SM and TIC to mental health nurses and allied health professionals …. [and] for staff to translate newly acquired  knowledge into practice by adopting SM and TIC strategies^B^, with a view to these becoming embedded as part of routine care in mental health service delivery^C^”  To reduce the use of restrictive interventions^A^ | - Sensory modulation (SM) - Trauma-informed-care (TIC) | - SM: “providing consumers with access to a range of sensory modalities suitable for their particular sensory needs, to help them to self-regulate their states of emotional arousal.” - TIC “A framework which acknowledges the impact of physical, psychological and sexual trauma on a person's mental health and strives to avoid any further trauma that may be caused in the delivery of mental health care” | - SM: Many services established sensory rooms and equipment - TIC: Not described | | |
| Sensory Room/Occupational Therapy (OT) Consultation/Sensory Motor Arousal Regulation Treatment (SMART) | | Warner/2013 | To treat affect and behavioral dysregulation^C^  Cohannet Academy: “To help develop safe and effective strategies^C^ for residents to manage and regulate physiological and emotional experiences”  OTA-GLC: “To address sensory modulation difficulties for their students” ^C^  “The aim of the GLC program is to better understand the needs of these students, and to develop a direct care, sensory integration-based model for the students who are most in need of support” ^C^  SMART: “To add power to a trauma psychotherapy by more effectively addressing the problems of affect and behavioral dysregulation which disrupt the daily lives of these traumatized children and adolescents and challenge their caregivers aand psychotherapists^C^” | - Cohannet Academy:   - Overarching ARC framework   - Individualized sensory diet   - Sensory Room: The “Getaway”   - The “Comfort Zone” - OTA Watertown:   - On site occupational therapy consultation   - Structural changes to unit   - Direct Occupational therapy intervention and referrals with Ayres Sensory Integration - OTA: Gifford   - OT room for use with multidisciplinary staff - OTA:GLC - Sensory comfort zones - Appropriate, sensory-modulating activities - Referral to OT - Sensory Motor Arousal Regulation Treatment (SMART) - The SMART Therapy room | - “Sensory Integration, a specialization within occupational therapy provides knowledge of the sensory motor systems and strategies for sensory modulation that addresses arousal regulation, which underlies this dysregulation.” | Cohannet Academy:   - “These tools are utilized on a voluntary basis by residents, and are available at most times of their day.” - Sensory diets: “staff began working with each resident individually to develop her own “sensory diet”…the purchase of “sensory tool boxes” for each resident to have in her room to access when dysregulated…This process starts at the pre-admission meeting…to find what is currently helpful. Some of the items are immediately purchased so on the day of admission they are available“ - The “Getaway” “space was used for group therapies, and individual and family sessions” - “Comfort Zone”: “Staff regularly sit with a resident and practice up and down regulation strategies… as a means to rehearse and utilize sensory interventions. There are other times the staff find residents need more outlets for managing energy… For some of these occasions, residents will wear ankle and wrist weights and skateboard or rollerblade up and down the hallways”   OTA Watertown-Brandon   - “When students require spaces for regulation …quiet rooms are available   …The quiet rooms are provided with mats for the floors… bean bag chairs, manipulative tactile and proprioceptive materials, and a chinup bar for older students… The Brandon students helped choose the wall colors of sand and blue to simulate a “calm beach” scene. The OT works with… staff to help in the selection of … activities and environments, and to make changes as student needs change.”   - “The occupational therapists provide one-to-one and small group sessions in the OT room, as well as consultation during activities of daily living …. The OT room is 14’ by 20’ and is equipped with both suspended swings as well as non-suspended equipment to address sensory integration issues.”   OTA: Gifford   - “The OT room was outfitted as a sensory motor area, with equipment provided for motor exploration and regulation of arousal…The room included equipment to address the body senses such as weighted blankets, Bosu balls for jumping and balancing, multiple crash cushions, large truck tire inner tubes for stacking to create both hide-aways and targets for jumping into, accordion mats for creating private spaces and for receiving whole body self-directed “squeezes”.” - “Following training by the OT… staff began to take children for “sensory break” times in the OT room during which students selected (often with assistance) materials and activities to help them regain or retain an arousal state that would allow them to participate in … aspects of the school day... Psychotherapists also began using the room…for conducting psychotherapy sessions”   OTA:GLC   - “GLC has referred students…to OTA-Watertown for in depth sensory integration-based OT evaluations … and then addressed through on site OT consultation and/or off-site direct OT intervention at OTA-Watertown”   SMART:   - “The outpatient clinic room is approximately 14’ by 17’ wide, allowing sufficient space for motor exploration. A gym mat was installed on the floor and movable 3’ × 5’ gym mats were utilized for safety, protection against the walls, and construction of smaller spaces for children to go into. Simple equipment, commonly found in gyms and play spaces, such as physioballs, a mini-trampoline, a low balance beam, a balance board, a tunnel, stepping stones, and blankets, as well as equipment commonly found in sensory integration based OT rooms such as large crash pillows filled with chunks of foam, a “walrus” air pillow, pieces of spandex and spandex body socks, a sensory shaker (bag of balls to climb inside) and 10 and 20 pound weighted blankets were included in the room… Adaptations of the space to meet adolescent needs are ongoing…importantly, a videotaping system was installed so that sessions could be taped for therapist training and supervision, parent guidance, SMART team learning, and model development.” - “All of the equipment in the SMART rooms is available for engagement.” - “With the therapist’s support, the adolescents explore [the room]… In some cases, they use the equipment and the movement afforded as an avenue and support to talk to the therapist about their daily lives as well as their traumatic experiences…In other instances, the equipment offers a non-verbal way to address difficult interpersonal dynamics.” | | |
| **Structured Psychotherapy**  **for Adolescents Responding to Chronic Stress (SPARCS)** | |  |  |  |  |  | | |
| SPARCS | | Habib/2013 | “To address the needs of adolescents who have witnessed, or directly experienced, repeated or multiple forms of violence”^C^  “Targets the emotional, social, and behavioral difficulties resulting from multiple exposures to violence”  “To help adolescents find the wisdom in their responses, support skills they already possess, and foster new ways of coping”^E^ | - SPARCS curriculum (16 session, manually-guided group intervention), with core skills based on the 4 Cs:   - Cultivating Awareness (of Self and Other)   - Coping Effectively   - Connecting with Others   - Creating Meaning - The “other 23 hours”: a milieu-based approach | - Based on complex trauma treatment principles and is grounded the following evidence-based interventions:   - Dialectical Behavior Therapy for Adolescents   - TARGET   - Trauma and Grief Component Therapy for Adolescents - Developmentally-sensitive, strengths-based, and present-focused | - Groups: approximately 1 hour in length, over 16-20 weeks | | |
| **Trauma Affect Regulation: Guide for Education and Therapy (TARGET)** | |  |  |  |  |  | | |
| TARGET and environmental modifications | | Marrow/ 2012 | To improve youth management and treatment outcomes ^E^  To reduce youth post traumatic stress symptoms ^E^, youth threats toward staff, and seclusion and restraint rates^A^  To provide training for staff on childhood traumatic stress^B^  To help staff problem solve effective ways to intervene with youth^B^  To enhance the unit environment by decreasing noise and providing safe places to practice skills^F^ | - 1- General trauma training for staff* - 2- TARGET (a 10-session manualized treatment and prevention intervention for traumatized adolescents and adults). TARGET is comprised of three main therapeutic components:   - Education   - Teaching and guided practice of the FREEDOM skills   - An experiential exercise where the client makes a timeline of their life - 3-Environmental modifications* | - “TARGET is designed to maximize a person’s awareness of the present moment, thereby reducing mental health symptoms commonly associated with trauma, such as rumination, panic, or dissociation” | - TARGET: 10 session manualized treatment, reinforced in 24/7 milieu environment - FREEDOM skills are integrated in the 24/7 milieu | | |
| **Trauma-Informed Psychiatric Residential Treatment (TI-PRT)** | |  |  |  |  |  | | |
| TI-PRT | | Boel-Studt/2017 | To create a supportive, therapeutic environment^F^  To achieve greater reductions in youth’s functional impairment^E^  To reduce restraints and seclusion^A^  To discharge youth in fewer months  To increase discharges to community-based placements^E^ | TI-PRT Enhancements (to traditional PRT)  •Trauma-focused individual therapy—EMDR or TF-CBT  •Trauma orientation/ongoing training  •Safety planning/ documentation  •Mission/shadow mission  •Daily member (client/staff) check-ins  •Family/caregiver education  •Trauma recovery group-based curriculum | - The trauma recovery curriculum is based on a combination of trauma treatments. - The youth are consistently   exposed to prosocial and adaptive processes versus power and control. | - Youth participate in a trauma recovery group-based curriculum 2 times per week. The groups are led by staff who are trained in the curriculum and are comprised of approximately 8–10 youth matched by age. - Member check-ins occur daily | | |
| **Trauma-Systems Therapy (TST)** | |  |  |  |  |  | | |
| Trauma Systems Therapy (TST) | | Brown/2013  Murphy/ 2017  Redd/2017 | “Provides both an organizing framework for identifying and coordinating the different service elements^C^ [in working with children with traumatic stress], as well as a clinical model that describes exactly what providers do^B^…” [Brown/2013]  “KVC selected TST with a goal of increasing child well-being, placement stability, and timeliness of permanency^E^ by implementing the approach with fidelity in all KVC service systems” [Redd/2017] | - “Dual emphasis on the emotional/behavioral functioning of the youth and the impact of the social environment” [Brown/2013]  1. Accurate Assessment: "Repeatedly assessing children's emotional and behavioral regulation capacity and the functioning of children's social environment” (including caregiver involvement and environment) [Murphy/2017]. “Moment by moment assessments” [Brown/2013] 2. Treatment (what providers do) 3. Organizational model for identifying and coordinating different service elements 4. Adaptations of TST to Residential Settings:    - A common language of care    - A focus on the social environment of the milieu (as well as caregivers – Ready Set Go module)    - A vehicle to integrate care across staff and team members    - Involving/training* the child’s community care team (therapists, case managers, foster and birth parents) [Redd/2017] | - “The primary clinical innovation that encapsulates TST is the concept of the trauma system”:   - “A traumatized child who has difficulty regulating emotional states”   - “A social environment or system of care that is not able to help the child regulate these emotional states” - “TST recognizes trauma as a barrier to children's self-regulation that needs to be addressed before children can recognize and deal with trauma through cognitive behavioral therapy and other treatments. It assumes that the “triggers” in children's environments causing “fight, flight or freeze” behaviors must be reduced or neutralized to foster a feeling of safety in children, which then permits them to recognize and deal with their trauma.” [Redd/2017] | - Each service is provided by separate clinicians who serve on a multi-specialty TST team - Assessment: at intake and repeated frequently | | |
| **Uncategorized TIC Programs** | |  |  |  |  |  | | |
| Gender-Specific and Trauma-Informed Training Curriculum | | Crable/2013 | “To increase awareness of best practice interventions; improve understanding of components of trauma-informed care; improve engagement skills with youth; improve emotional and physical boundaries with youth; improve understanding of cycle of sexual retraumatization”^B^ | - Training curriculum consisting of eight modules: *   - In depth look at what is trauma   - Risk and protective factors   - Review of trauma-informed interventions   - Overview of trauma reactions   - Signs and symptoms of trauma   - Tools for engaging and helping traumatized youth   - How empowerment is instrumental to healing   - Tips for creating therapeutic milieus | - “Without adequate training counselors will not be prepared to understand the family dynamics of sexual abuse and most importantly how to identify victims of childhood sexual abuse.” | Not described** | | |
| Trauma-Informed Approach (TIA) | | Craig/2018 | To “minimize the chance for everyday operations to traumatize or re-traumatize that client during service delivery.”  “To reduce or eliminate restraint and seclusion”^A^ | - Response blocking - Knowledge of behavioural intent and client needs - Creative solutions as alternatives to restraint and seclusion. | - “TIA is a multilevel approach to treatment that begins with physical and emotional safety provided by an adult caregiver …With the presentation of a safe environment, children will be open to altering behavior, considering new ideas, and accepting help instead of worrying about their survival.” | - Treatment planning assessment performed by newly formed treatment team | | |
| Trauma-Informed Care (TIC) Program | | Jacobowitz/ 2015 | NR | NR | NR | Not described** | | |
| Trauma-Informed Child Welfare Service (CWS)/ The Connecticut Collaborative on Effective Practices for Trauma (CONCEPT) | | Lang/2013 | “The broad goal of CONCEPT is to create a more trauma informed  CWS that integrates research and best practices on childhood trauma and ultimately results in improved identification, case planning, and service delivery for children and  families”^C^  “The long-term goal of CONCEPT is to implement trauma screening for all children receiving in- home or out-of-home CPS and to embed screening data into the Statewide Automated Child Welfare Information System (SACWIS)”^C^ | - Trauma screening - Availability of EBP treatments - Trauma-informed policy and practice guide revisions* (including considerations for assessing and supporting caregivers) | - “Screening children for PTEs [potentially traumatic events] and traumatic stress reactions is a key component of trauma-informed care and is the primary strategy…for ‘‘recognizing’’ children suffering from trauma.” | Not described | | |
| Trauma-Informed Care (TIC) Training Program | | Williams/ 2017 | “To enable the adoption of trauma informed approaches throughout public mental health and alcohol and other drug services in Western Australia”^C^ | - Training program* | NR | Not described** | | |

*We (the review authors) perceived these components as implementation strategies

**See Table S2 for summary of implementation strategies

**AIMS Footnotes Legend:**

| AIMS Category legend | Number of interventions (N=21) |
| --- | --- |
| A To reduce use of restraints/seclusions/incidence of critical events | 7 |
| B To change staff attitudes, practices | 11 |
| C To increase/change available assessments and treatments/ coordinate care | 10 |
| D To support staff wellness/increase staff self-capacities | 4 |
| E To increase patient capacity/improving patient outcomes | 8 |
| F To change culture | 12 |
